# ARID5B‐mediated LINC01128 epigenetically activated pyroptosis and apoptosis by promoting the formation of the BTF3/STAT3 complex in β2GPI/anti‐β2GPI‐treated monocytes

**DOI:** 10.1002/ctm2.1539

**Published:** 2024-01-15

**Authors:** Yuan Tan, Jiao Qiao, Shuo Yang, Qingchen Wang, Hongchao Liu, Qi Liu, Weimin Feng, Boxin Yang, Zhongxin Li, Liyan Cui

**Affiliations:** ^1^ Institute of Medical Technology Peking University Health Science Center Beijing China; ^2^ Department of Laboratory Medicine Peking University Third Hospital Beijing China; ^3^ Core Unit of National Clinical Research Center for Laboratory Medicine Peking University Third Hospital Beijing China

**Keywords:** apoptosis, APS, ARID5B, epigenetics, LINC01128, pyroptosis, thrombosis

## Abstract

**Background:**

Alterations of the trimethylation of histone 3 lysine 4 (H3K4me3) mark in monocytes are implicated in the development of autoimmune diseases. Therefore, the purpose of our study was to elucidate the role of H3K4me3‐mediated epigenetics in the pathogenesis of antiphospholipid syndrome (APS).

**Methods:**

H3K4me3 Cleavage Under Targets and Tagmentation and Assay for Transposase‐Accessible Chromatin were performed to determine the epigenetic profiles. Luciferase reporter assay, RNA immunoprecipitation, RNA pull‐down, co‐immunoprecipitation and chromatin immunoprecipitation were performed for mechanistic studies. Transmission electron microscopy and propidium iodide staining confirmed cell pyroptosis. Primary monocytes from patients with primary APS (PAPS) and healthy donors were utilised to test the levels of key molecules. A mouse model mimicked APS was constructed with beta2‐glycoprotein I (β2GPI) injection. Blood velocity was detected using murine Doppler ultrasound.

**Results:**

H3K4me3 signal and open chromatin at the *ARID5B* promoter were increased in an in vitro model of APS. The epigenetic factor ARID5B directly activated LINC01128 transcription at its promoter. LINC01128 promoted the formation of the BTF3/STAT3 complex to enhance STAT3 phosphorylation. Activated STAT3 interacted with the *NLRP3* promoter and subsequently stimulated pyroptosis and apoptosis. ARID5B or BTF3 depletion compensated for LINC01128‐induced pyroptosis and apoptosis by inhibiting STAT3 phosphorylation. In mice with APS, β2GPI exposure elevated the levels of key proteins of pyroptosis and apoptosis pathways in bone marrow‐derived monocytes, reduced the blood velocity of the ascending aorta, increased the thrombus size of the carotid artery, and promoted the release of interleukin (IL)‐18, IL‐1β and tissue factor. Patients with PAPS had the high‐expressed ARID5B and LINC01128, especially those with triple positivity for antiphospholipid antibodies. Moreover, there was a positive correlation between ARID5B and LINC01128 expression.

**Conclusion:**

This study indicated that ARID5B/LINC01128 was synergistically upregulated in APS, and they aggravated disease pathogenesis by enhancing the formation of the BTF3/STAT3 complex and boosting p‐STAT3‐mediated pyroptosis and apoptosis, thereby providing candidate therapeutic targets for APS.

**Highlights:**

- The H3K4me3 mark and chromatin accessibility at the *ARID5B* promoter are increased in vitro model mimicked APS.
- ARID5B‐mediated LINC01128 induces pyroptosis and apoptosis via p‐STAT3 by binding to BTF3.
- ARID5B is high‐ expressed in patients with primary APS and positively correlated with LINC01128 expression.
- OICR‐9429 treatment mitigates pyroptosis and related inflammation in vivo and in vitro models mimicked APS.

## Graphical abstract

This study demonstrates that the H3K4me3 mark and chromatin accessibility at the ARID5B promoter are increased in vitro model mimicked APS. ARID5B‐mediated LINC01128 induces pyroptosis and apoptosis via p‐STAT3 by binding to BTF3. ARID5B is high‐expressed in patients with primary APS and positively correlated with LINC01128 expression. OICR‐9429 treatment mitigates pyroptosis and related inflammation in vivo and in vitro models mimicked APS.

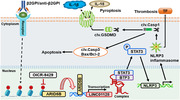

## 1 INTRODUCTION

Antiphospholipid syndrome (APS) belongs to a category of autoimmune diseases and accompanies with the formation of thrombus, adverse pregnancy outcomes (APOs) and persistent positivity for antiphospholipid antibodies (aPLs).^1^ aPLs are disease indicators and play a primary pathogenetic role in APS by interacting with target receptors on the cell membrane. In addition, they are involved in activating endothelial cells, neutrophils, platelets and monocytes and releasing inflammatory mediators and coagulation factors.^2^, ^3^ Although in vitro studies and animal experiments have revealed the involvement of epigenetics in the pathogenesis of APS, including long non‐coding RNAs (lncRNAs) and cytosine‐phosphate‐guanine (CpG) methylation at the promoters of genes,^4^, ^5^, ^6^ the mechanisms of histone modification in its occurrence and development remain to be elucidated.^3^, ^7^

Trimethylation of histone 3 lysine 4 (H3K4me3) is a major histone modification, resulting in dynamic alterations of chromatin accessibility and the expression of inflammation‐related genes, suggesting its potential role in the pathogenesis of APS.^8^, ^9^ The interaction of WD repeat domain 5 (WDR5), a H3K4 presenter that forms the COMPASS complex together with ASH2L, DPY30 and RBBP5,^10^ with H3K4 methyltransferase MLL1 mainly catalyses the deposition of the H3K4me3 mark.^11^ OICR‐9429, a novel WDR5 inhibitor competitively disrupts the MLL1–WDR5 complex via binding the central peptide‐binding pocket of WDR5, thereby resulting in the specific reduction of H3K4me3 enrichment but not WDR5 expression.^12^, ^13^ Therefore, inhibition of MLL1–WDR5 interaction by OICR‐9429 is capable of blocking H3K4me3‐mediated gene transcription.

Pyroptosis is a unique proinflammatory form of cell death that exerts a crucial function in aggravating the development of multiple autoimmune diseases, such as rheumatoid arthritis,^14^ multiple sclerosis,^15^ systemic lupus erythematosus^16^ and APS.^17^, ^18^ Pyroptosis is first identified in macrophages, and the assembly of Nod‐like receptor family pyrin domain‐containing 3 (NLRP3) inflammasome is a key event,^19^, ^20^ which is associated with apoptosis.^21^ The activated NLRP3 inflammasome facilitates the production of bioactive interleukin (IL)‐18 and IL‐1β, and cleaves gasdermin D (GSDMD) into a segmented N‐terminal fragment and forms a pyroptotic pore.^22^, ^23^ Secretory IL‐18 and IL‐1β result in a proinflammatory response and cell injury.^24^ Evidence has revealed that the beta2‐glycoprotein I (β2GPI)/anti‐β2GPI complex can induce pyroptosis of endothelial cells and neutrophils by increasing the expression of NLRP3, cleaved (clv.) Casp1 and clv. GSDMD in APS.^17^, ^18^ Nevertheless, the underlying mechanisms of β2GPI/anti‐β2GPI in mediating monocyte pyroptosis are not fully understood.

Expression profiles of lncRNAs are dysregulated in monocytes isolated from patients with primary APS (PAPS) compared to that in healthy controls.^4^ The length of lncRNAs is more than 200 nucleotides, they have an essential function in regulating cell growth, death and relevant inflammation through influencing the epigenetic changes, transcriptomic levels and posttranscriptional modification.^25^ Recently, studies have suggested that lncRNAs are implicated in regulating macrophage pyroptosis.^26^ For instance, LINC01272 was upregulated in nicotine‐induced pyroptosis of macrophages through activating transcription factor KLF6‐mediated GSDMD‐N and NLRP3 expression.^27^ Nevertheless, the impact of lncRNAs on β2GPI/anti‐β2GPI‐induced pyroptosis of monocytes and macrophages in APS remains to be elaborated.

In this study, we performed Cleavage Under Targets and Tagmentation (CUT&Tag) and Assay for Transposase‐Accessible Chromatin using sequencing (ATAC‐Seq) for H3K4me3 to reveal the epigenetic features in an in vitro monocyte model mimicked APS. The data showed that the specific H3K4me3 mark and open chromatin at the *ARID5B* promoter were enhanced. ARID5B positively regulated LINC01128 expression in pyroptotic monocytes induced by β2GPI/anti‐β2GPI as revealed by epigenetic analysis and cell culture experiments. Subsequently, we explored the regulatory mechanisms of LINC01128 in β2GPI/anti‐β2GPI‐induced pyroptosis of monocytes and analysed the correlation between ARID5B and LINC01128 along with clinicopathological characteristics in patients with PAPS. Finally, we validated the pathological changes in vivo in mice with APS and the levels of ARID5B, LINC01128 and downstream targets.

## 2 MATERIALS AND METHODS

### 2.1 Cell culture

Tohoku Hospital Pediatrics‐1 (THP‐1), a human monocytic cell line, was obtained from Shanghai Institutes of Biological Sciences and maintained in an incubator with 5% CO_2_ at 37°C with RPMI 1640 containing 10% foetal bovine serum (Gibco). Transfected cells were selected using puromycin (Sigma–Aldrich). After starving for 16 h, THP‐1 cells were exposed to OICR‐9429 (20 μM, HY‐16993, MedChemExpress) for 24 h and stimulated with the immune complex (IC: β2GPI [100 μg/mL, 11221‐H08H, Sino Biological Inc.]/anti‐β2GPI [10 μg/mL, 11221‐R003, Sino Biological Inc.]) for 4 h for RNA and DNA analyses and 6 h for protein analysis.

Peripheral blood mononuclear cells from three healthy donors (HDs) were extracted using 1.077 g/mL Lymphoprep density gradient medium (StemCell Technologies). APC anti‐human CD14 antibody (301807, BioLegend) and Human Monocyte Isolation Kit (#19359, StemCell Technologies) were used to isolate primary monocytes. After overnight culture, the non‐adherent monocytes were removed. Then, starving cells for 16 h, pretreating with OICR‐9429 for 24 h, and stimulating with IC for 4 h for RNA and DNA analyses and 6 h for protein analysis.

### 2.2 Lentivirus and plasmid transfection

Small interfering RNAs (siRNAs) against BTF3 (siBTF3#1: GCCGAAGAAGCCUGGGAAUCA; siBTF3#2: GCAGGCACAAGUGCGCAUUTT), STAT3 (siSTAT3#1: GUUGAAUUAUCAGCUUAAA; siSTAT3#2: CAUCUGCCUAGAUCGGCUA), NLRP3 (siNLRP3: CAACAGGAGAGACCUUUAU) and small interfering negative control (siNC) were constructed by GenePharma Technologies. Smart silencer of LINC01128 was purchased from RIBOBIO, and the overexpression (OE) plasmid of LINC01128 (OE‐LINC01128) was purchased from GeneKai. Cells were transfected at 50% confluency using jetPRIME in vitro siRNA transfection reagent (#114‐01, Polyplus).

ARID5B shRNA (shARID5B#1: GCCTTCAAAGAGAACCATTTA; shARID5B#2: CTACACCTGTAGGAAGTTCAT), scrambled negative control (shNC), OE‐ARID5B, OE‐STAT3 and negative control (OE‐NC) lentiviruses were constructed by GeneKai. After infecting with lentiviruses, cells were selected using puromycin (3 μg/mL). For co‐transfection, transfecting THP‐1 cells with the LINC01128 overexpression plasmid for 24 h, then performing a 48 h of infection with ARID5B shRNA or BTF3 siRNA.

### 2.3 Western blotting

Cells were harvested for extracting proteins with RIPA lysis (Beyotime). After collecting supernatant, protein quantification was conducted by the BCA Kit (Beyotime). Separating proteins using SDS‐PAGE and transferring proteins using a polyvinylidene fluoride membrane, subsequently the membrane was blocked with 5% defatted milk. Next, incubating with the specific primary antibodies: anti‐STAT3 (1:400, sc‐8019, Santa Cruz Biotechnology), anti‐ARID5B (1:500, NBP1‐83622, Novus), anti‐cleaved GSDMD (mouse, 1:1000, #10137, CST), anti‐cleaved GSDMD (human, 1:1000, #36425, CST), anti‐NLRP3 (1:1000, PA5‐79740, Invitrogen), anti‐p‐STAT3 (1:1000, #6774, CST), anti‐cleaved caspase 1 (human, 1:1000, #4199, CST), anti‐ASC (1:1000, ab155970, Abcam), anti‐BTF3 (1:400, sc‐166093, Santa Cruz Biotechnology), anti‐cleaved caspase 1 (mouse, 1:1000, #89332, CST), anti‐cleaved IL‐1β (mouse, 1:1000, #63124, CST), anti‐cleaved caspase 3 (1:1000, #9661, CST), anti‐Bcl‐2 (1:500, sc‐7382, Santa Cruz Biotechnology), anti‐cleaved IL‐1β (human, 1:1000, #83186, CST), anti‐Bax (1:500, sc‐7480, Santa Cruz Biotechnology) and anti‐β‐actin (1:3000, 66009‐1‐Ig, Proteintech). After incubating with goat anti‐mouse IgG H+L (horseradish peroxidase, (HRP)) (1:5000, ab205719, Abcam) or goat anti‐rabbit IgG H+L (HRP) (1:5000, ab6721, Abcam) secondary antibodies, the intensity of signals was measured using the enhanced ECL Kit (Millipore).

### 2.4 Real‐time quantitative PCR

Following the reagent's protocol, cells were lysed using the TRIzol to extract RNA (15596026, Invitrogen). Hifair III 1st Strand cDNA Synthesis SuperMix (11141ES60, Yeasen Biotec) was utilised for reversely transcribing RNA (1 μg) into complementary DNA. Hieff qPCR SYBR Green Master Mix (11184ES08, Yeasen Biotec) was applied for the quantification of target genes. The primers are concluded in Supporting Information 1.

### 2.5 Subcellular fractionation assay

RNA locating in nucleus and in cytoplasm were separated using cytoplasmic and nuclear RNA purification Kit (NGB‐21000, Norgen Biotek) following the Kit's protocol. RNA quantification in nucleus or cytoplasm of U6, LINC01128 and β‐actin was assessed by real‐time quantitative PCR (RT‐qPCR).

### 2.6 Luciferase activity reporter assay

The binding site of ARID5B at the promoter of *LINC01128* was potentially estimated by http://bioinfo.life.hust.edu.cn/hTFtarget#!/website. Wild‐type (WT‐LINC01128) or mutant LINC01128 promoter (Mut‐LINC01128) constructs were synthesised and cloned into the GV238 vector to obtain the corresponding plasmids. After co‐transfecting 293T cells with WT‐LINC01128 or Mut‐LINC01128 plasmids and OE‐ARID5B or OE‐NC plasmids, the Dual‐Luciferase Reporter Assay System (E1910, Promega) was used to evaluate the relative luciferase activity.

### 2.7 CUT&Tag

The Hyperactive Universal CUT&Tag Assay Kit (TD903, Vazyme) was employed for performing CUT&Tag. Monocytes or THP‐1 cells (1 × 10^5^) were mixed with active ConA Beads. Cells were incubated with the primary and the secondary antibody (1:100), then the ConA bead complex was incubated with pA/G‐Tnp to obtain fragmented DNA. Target DNA was extracted using DNA extraction beads, and a DNA spike was added to normalise the sequencing data. The library was constructed using the TruePrepTM Index Kit V2 (TD202, Vazyme). VAHTS DNA Clean Beads (#N411, Vazyme) were used to purify the PCR products, and library concentration and quality were evaluated using Qubit fluorometric quantitation. Finally, DNA was subjected to paired‐end Illumina NovaSeq 6000 sequencing, or qPCR, and hg38 was used as the reference genome for sequencing analysis.

### 2.8 ATAC‐seq

The TruePrepTM DNA Library Prep Kit V2 (TD501, Vazyme) was used to perform ATAC‐Seq. Briefly, 5 × 10^4^ monocytes, or THP‐1 cells, were lysed to collect the cell nuclei. After purifying the fragmented DNA using VAHTS DNA Clean Beads (#N411, Vazyme), library construction was performed using the TruePrepTM Index Kit V2 (TD202, Vazyme). The purified PCR products were assessed using Qubit fluorometric quantitation, and paired‐end Illumina NovaSeq 6000 sequencing and bioinformatics analysis were performed. hg38 was used as the reference genome for sequencing analysis.

### 2.9 Transmission electron microscopy

Pyroptosis was detected by transmission electron microscopy (TEM).^28^, ^29^ THP‐1 cells (1 × 10^6^) were collected for fixation with 2.5% glutaraldehyde for 40 min and then placed at 4°C for overnight incubation, followed by the second fixation with 1% osmic acid for 1.5 h. Performing gradual dehydration in 30%, 50%, 70%, 90%, and 100% acetone, then embedding the cell mass with ethoxyline resin. Sectioning the cell samples into 50 nm each slice and incubating with 4% uranyl acetate–lead citrate, and autophagic vacuoles were subsequently observed by TEM (JEM1400PLUS).

### 2.10 Cell Counting Kit‐8 assay

THP‐1 cells or monocytes were plated in a 96‐well plate at the density of 10 000 cells/well, and cells were administrated with OICR‐9429 for 24 h and exposed to IC for 4 h. Afterwards, Cell Counting Kit‐8 (CCK‐8) reagent (10 μL) (GK10001, GLPBIO) was added and mixed with the cells for a 1 h incubation at 37°C. The optical density was tested at 450 nm.

### 2.11 Lactate dehydrogenase assay

The Cytotoxicity LDH Assay Kit (GK10003, GLPBIO) was employed to measure cell death by evaluating the levels of lactate dehydrogenase (LDH). The optical density was measured at 490 nm.

### 2.12 Immunofluorescence

Cells were harvested for fixation in 4% formaldehyde and permeabilisation in .3% Triton X‐100, and then blocked with 2% bovine serum albumin (BSA). After incubating with the primary antibody overnight and goat anti‐mouse IgG H+L (Alexa Fluor 488) (1:300, ab150013, Abcam) or goat anti‐rabbit IgG H+L (Alexa Fluor 555) (1:300, ab150078, Abcam) secondary antibody at 37°C for 30 min, 4,6‐diamino‐2‐phenyl indole (DAPI) (28718‐90‐3, Sigma–Aldrich) was used for staining nuclei for 5 min. Subsequently, the slices were coated with an anti‐quenching reagent, and fluorescent signals were evaluated using a fluorescence microscope (Leica).

### 2.13 Flow cytometry analysis

The Apoptosis Detection Kit (C1062, Beyotime) detected pyroptosis in THP‐1 cells or monocytes following the kit's instructions. Cells were harvested and resuspended with 1× Annexin V‐FITC binding buffer. Thereafter, the cell samples were darkly stained with Annexin V‐FITC and propidium iodide (PI) for about 15−20 min. The apoptotic rate of cells was evaluated using C6 Flow Cytometer system (BD Biosciences).

### 2.14 Fluorescence in situ hybridisation

Fluorescence in situ hybridisation (FISH) was conducted using the FISH Kit (RiboBio) following the kit's instructions.^30^, ^31^ Biotin‐labelled LINC01128 probe (5′‐GGAGUUCUUCAUUCCCACAUCUGUGGACCUUAAAAUCCUACCGUUGAGUGCCUGCCUUGGAUCAGCAGUCAGUUCGUUACCUUGGUUCUCAGACGAUGCUUGCAACAACGC‐3′) was designed by GZSCBIO. Cell slides were prepared for fixation in 4% paraformaldehyde and permeabilisation in .5% Triton X‐100, then blocking with prehybridisation buffer and darkly staining cells with the LINC01128 probe for overnight incubation at 37°C. Ultimately, fluorescence was visualised using a confocal microscope (LSM780, Zeiss).

### 2.15 RNA immunoprecipitation

As reported previously, the EZ Magna RIP Kit (Millipore) was applied for the RNA immunoprecipitation (RIP) assay.^30^ Cells (1 × 10^8^) were harvested for lysing with RIP lysis. After washing with wash buffer, protein A/G magnetic beads were pre‐conjugated with anti‐BTF3 (sc‐166093, Santa Cruz Biotechnology), anti‐STAT3 (sc‐8019, Santa Cruz Biotechnology) or anti‐IgG (A7001, Beyotime) antibodies to form the complexes of protein A/G magnetic bead antibody. Then, mixing the cell lysate with the complexes for overnight incubation at 4°C, the purified RNA products were detected by RT‐qPCR using proteinase K buffer.

### 2.16 Chromatin isolation by RNA purification

Chromatin isolation by RNA purification (ChIRP) was conducted as reported previously.^30^ ChIRP probes against LINC01128 (Supporting Information 2) and LacZ were constructed by GZSCBIO. THP‐1 cells (1 × 10^8^) were cross‐linked with 1% glutaraldehyde, de‐crosslinked with .125 M glycine solution and sonicated. The sonicated cell lysate was hybridised with the biotinylated DNA probe mixture for LINC01128 and incubated at 4°C overnight. The probes were extracted using streptavidin‐coated magnetic beads. Finally, the combined DNA was subjected to qPCR or Illumina NovaSeq 6000, and hg38 was used as the reference genome for sequencing analysis. The denatured proteins were analysed by western blotting.

### 2.17 RNA pull‐down

RNA pull‐down assay was conducted according to the previous reports.^30^ Briefly, the LINC01128 overexpression vector was designed by Sangon Biotech to serve as a template to amplify the LINC01128‐corresponding antisense and sense strands using the T7 promoter‐containing primers. Biotinylated LINC01128 was synthesised by the TranscriptAid T7 High Yield Transcription Kit (Thermo Fisher Scientific), which was subsequently incubated with streptavidin‐coated magnetic beads. The RNA pull‐down assay was conducted using the Magnetic RNA‐Protein Pull‐Down Kit (Pierce 20164, Thermo Fisher Scientific). Subsequently, the precipitated proteins were employed for silver staining, western blotting and mass spectrometry. The RNA–protein interaction probabilities generated by http://pridb.gdcb.iastate.edu/RPISeq/about.php (RNA–Protein Interaction Prediction, RPISeq) ranged from 0 to 1. Reaction probabilities were represented as values of random forest (RF) and support vector machine (SVM), and only those values where both RF >.5 and SVM >.5 were identified as ‘positive’, displaying that the possible interaction of protein with RNA.

### 2.18 Co‐immunoprecipitation

Briefly, THP‐1 cells were prepared for lysing using lysis buffer (P0013, Beyotime), and incubating the protein lysate with protein A/G plus agarose (sc‐2003, Santa Cruz Biotechnology) for 10 min to avoid non‐specific binding. After centrifugation, the protein supernatant was mixed with anti‐STAT3 (sc‐8019, Santa Cruz Biotechnology), anti‐BTF3 (sc‐166093, Santa Cruz Biotechnology) or anti‐IgG (A7001, Beyotime) antibodies for overnight incubation at 4°C. Next, adding protein A/G agarose for a 3 h incubation at 4°C. Finally, the protein complexes were eluted, then assessed using western blotting.

### 2.19 Chromatin immunoprecipitation assay

Chromatin immunoprecipitation (ChIP) was conducted according to the previous reports.^32^ Cells (2 × 10^7^) were prepared for cross‐linking in 1% paraformaldehyde, de‐crosslinking with .125 M glycine solution, and sonicating. After precipitating the immunocomplexes by incubating soluble chromatin with an anti‐p‐STAT3 (#6774, CST) antibody, ChIP DNA was identified using qPCR. Relative DNA enrichment was represented as ΔCt [normalised IP = (Ct [IP] – (Ct [input] – log2 [input dilution factor])), input dilution factor = 10, %Input = 2^(–ΔCt [normalised IP]) × 100%).

### 2.20 Mouse model of vascular APS

BALB/c mice (8–10 weeks old, female) were obtained from Beijing Vital River Laboratory Animal Technology and kept in a specific pathogen‐free (SPF) environment. Randomly dividing mice into three groups: negative control (NC) group (NC mice) injected with Freund's adjuvant (F5881, Sigma–Aldrich) containing BSA; β2GPI group (APS mice), intraperitoneally injected with Freund's adjuvant containing 100 μg β2GPI protein (11221‐H08H, Sino Biological Inc.) on days 0, 7 and 14^33^, ^34^, ^35^; and β2GPI plus OICR‐9429‐treated (OICR‐9429+β2GPI) group, exposed to 5 mg/kg of OICR‐9429 daily at day 15 for 7 consecutive days. Collecting blood samples from the inner canthus to evaluate the levels of anti‐β2GPI, IL‐1β, tissue factor (TF) and IL‐18 using ELISA on day 22. On day 28, blood velocity of the ascending aorta was detected using murine Doppler ultrasound. FeCl_3_ (10%) was applied to soak the Whatman filter paper (3 mm × 1 mm) and placed under the carotid artery for 5 min, after which the arteries were removed to assess the thrombus size using haematoxylin–eosin staining.

BALB/c mice were sacrificed, and their blood was rapidly harvested to detect activated partial thromboplastin time (APTT) and platelet count (PLT). In addition, collecting and washing femurs (thigh bones) with phosphate‐buffered saline. After cutting off the cartilage at both ends, the bone marrow fluid was collected from the bone marrow cavity using a 1 mL syringe. Marrow fluid was filtered with a 70 μm strainer to harvest marrow cells, then removing red blood cells with lysis buffer. Bone marrow‐derived monocytes were extracted using the specific Mouse Monocyte Isolation Kit (#19861, StemCell Technologies), and lysed for extracting protein and evaluating functional molecules.

### 2.21 ELISA

The levels of anti‐β2GPI (MEIMIAN, China), TF in mice serum (E‐EL‐M1163c), IL‐18 in mice serum (E‐EL‐M0730c), IL‐1β in mice serum (E‐MSEL‐M0003), IL‐1β in cell supernatant (E‐EL‐H0149c) and IL‐18 in cell supernatant (E‐EL‐H0253c) were assessed by elabscience ELISA Kits, then detecting the absorbance at 450 nm.

### 2.22 Participants

Sixty‐four adult patients with PAPS, between November 2022 and August 2023, were included in our study according to the Sydney classification criteria.^36^ Patients diagnosed with other autoimmune diseases, cancers or infectious diseases were excluded. Thirty‐two age‐, sex‐ and ethnicity‐matched HDs represented the control group. We examined the demographic, clinical and laboratory features, including age and gender, history of venous/arterial thrombosis and APOs, the levels of complement C3 and C4, PLT, normalised dilute Russell's viper venom time (dRVVT) and silica clotting time (SCT), and the titres of aCL and anti‐β2GPI (Supporting Information S3). The blood samples from patients with PAPS and HDs were collected to extract primary monocytes.

### 2.23 Statistics

All statistical analyses were represented as mean ± SD analysis using GraphPad Prism v8.0.1. The significant comparison between the two groups was calculated with Mann–Whitney *U*‐test or two‐tailed unpaired Student's *t*‐test demonstrated. Chi‐squared test was performed to compare categorical variables. *p*‐Values were expressed as: ^***^ *p* < .001, ^**^ *p* < .01, ^*^ *p* < .05 and ns, not significant.

## 3 RESULTS

### 3.1 H3K4me3‐mediated ARID5B expression at its promoter in an in vitro monocyte model of APS

**FIGURE 1:**
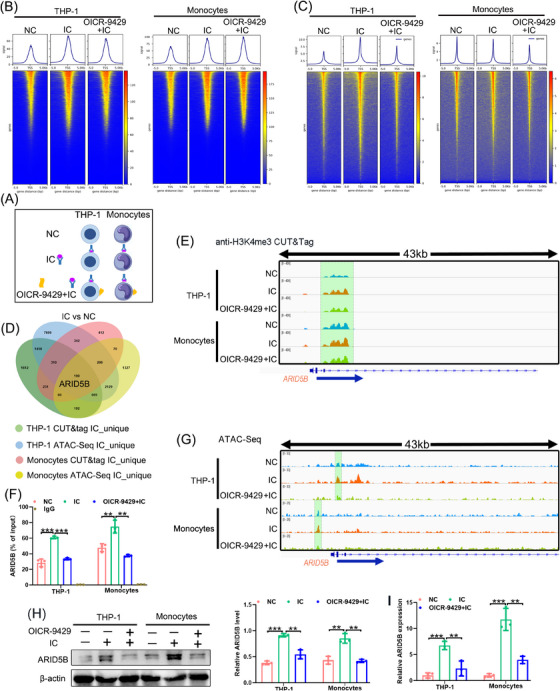
Trimethylation of histone 3 lysine 4 (H3K4me3)‐mediated ARID5B expression at its promoter. (A) Workflow of β2GPI/anti‐β2GPI immune complex (IC) treatment and OICR‐9424 exposure in an ex vivo monocyte or THP‐1 cell model that partially mimicked antiphospholipid syndrome (APS). (B) Heatmaps of H3K4me3 Cleavage Under Targets and Tagmentation (CUT&Tag) in an ex vivo THP‐1 cell and monocyte model of APS. (C) Heatmaps of Transposase‐Accessible Chromatin using sequencing (ATAC‐Seq) in an ex vivo THP‐1 cell and monocyte model of APS. (D) Venn diagram showed the intersection of the unique peaks (ARID5B) in the IC group compared to that in the negative control (NC) group between H3K4me3 CUT&Tag and ATAC‐Seq. (E) Integrative Genomics Viewer (IGV) and (F) quantitative PCR (qPCR) showed the relative enrichment levels of H3K4me3 at the promoter of ARID5B. (G) Chromatin accessibility at the ARID5B promoter was displayed using IGV. (H) The protein levels of ARID5B in the ex vivo model of APS were detected using western blotting. (I) Real‐time quantitative PCR (RT‐qPCR) determined the mRNA expression of ARID5B in the ex vivo model of APS. Data information: error bars represent the mean ± SD of at least three independent experiments. ^**^ *p* < .01; ^***^ *p* < .001. β2GPI, beta2‐glycoprotein I.

H3K4me3 at promoters is tightly correlated with the activation of gene expression,^37^ which is a predictor of chromatin accessibility,^38^ and can be reduced by OICR‐9429.^12^, ^13^ To investigate H3K4me3‐mediated chromatin dynamics in APS, we first established an ex vivo model partially mimicking APS by stimulating monocytes or THP‐1 cells with the β2GPI/anti‐β2GPI IC. THP‐1 cells and monocytes were divided into NC, IC and OICR‐9429+IC groups (Figure 1A). H3K4me3 CUT&Tag and ATAC‐Seq were performed together to determine the presence of active promoter regions and open chromatin. Results showed that active H3K4me3 and open chromatin regions were typically near transcriptional start sites (TSS) (Figure 1B,C). Compared to the NC group, we analysed the unique peaks in the IC group and further selected the intersection peaks from CUT&Tag and ATAC‐Seq data of THP‐1 cells and monocytes (Figure 1D). There were 190 intersecting unique peaks observed in both analyses (Figure 1D), they held more active H3K4me3 and more accessible chromatin.

Among these unique intersection peaks, the transcriptional upregulation of the epigenetic factor ARID5B has been identified in an in vitro model of APS.^5^ Furthermore, the H3K4me3 signal and open chromatin at the *ARID5B* promoter were dramatically augmented in the in vitro monocyte and THP‐1 models that partially mimicked APS and decreased after OICR‐9429 treatment (Figure 1E–G). Consistent with these results, western blotting and RT‐qPCR showed that ARID5B was upregulated in the IC group and downregulated in the OICR‐9429+IC group (Figure 1H,I). Therefore, ARID5B was the focus of subsequent experiments.

### 3.2 ARID5B transcriptionally activated LINC01128 expression

**FIGURE 2:**
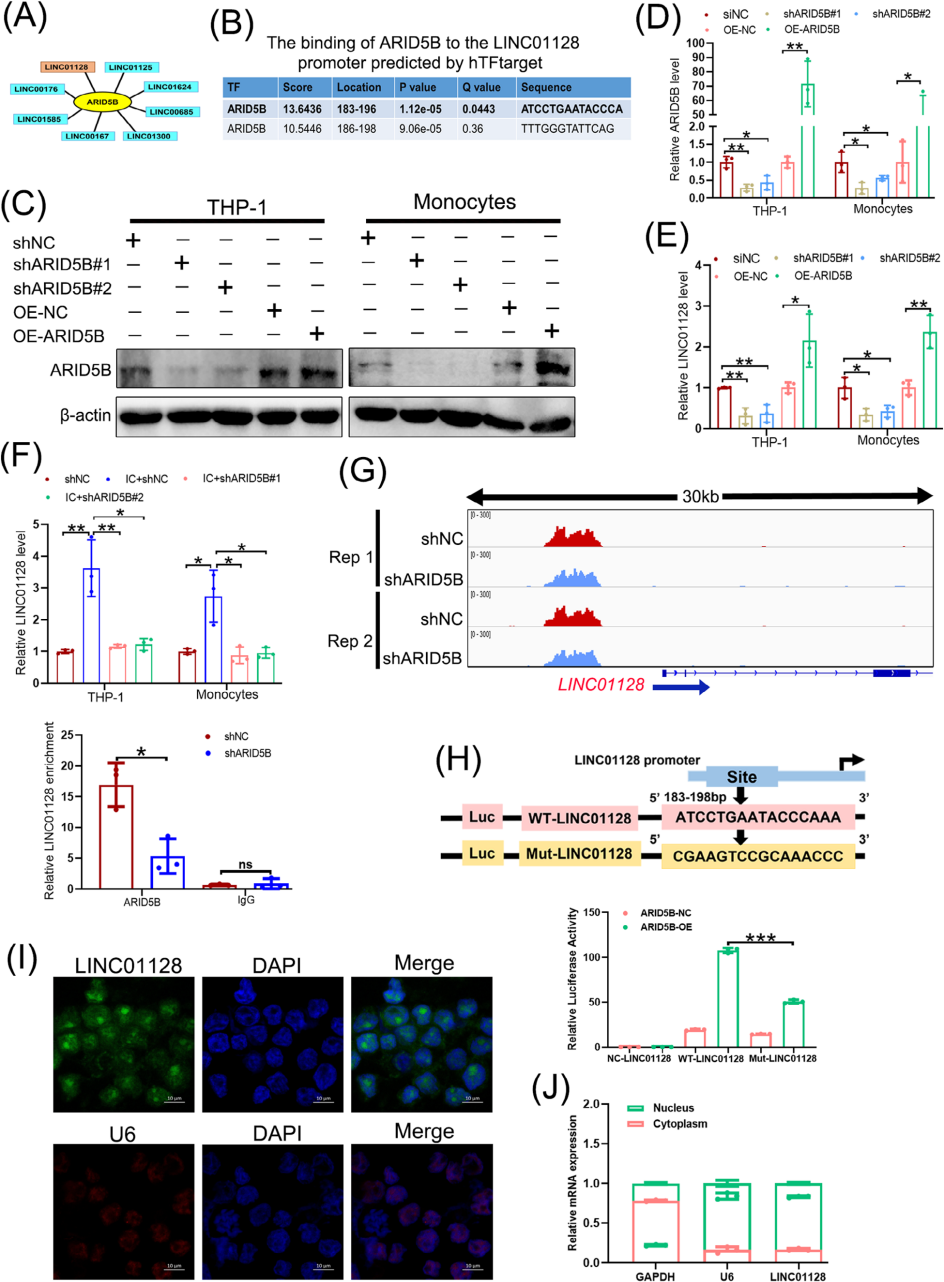
ARID5B transcriptionally activated LINC01128 expression. (A) Schematic representation of eight target long non‐coding RNAs (lncRNAs) of ARID5B in anti‐ARID5B Cleavage Under Targets and Tagmentation (CUT&Tag) in THP‐1 cells. (B) The two binding sites of ARID5B at the LINC01128 promoter were predicted using http://bioinfo.life.hust.edu.cn/hTFtarget#!/website. (C) Western blotting and (D) real‐time quantitative PCR (RT‐qPCR) analysis of ARID5B expression after transfection with two shRNAs (shARID5B#1 and shARID5B#2) or overexpression lentivirus in THP‐1 cells and monocytes. (E) RT‐qPCR analysis of LINC01128 expression after transfection with the above shRNAs or overexpression lentivirus. (F) RT‐qPCR analysis of LINC01128 expression after treatment with β2GPI/anti‐β2GPI immune complex (IC) in shARID5B‐THP‐1 cells. (G) Integrative Genomics Viewer (IGV) and quantitative PCR (qPCR) representation of anti‐ARID5B CUT&Tag at the *LINC01128* promoter in the scrambled negative control (shNC)‐ and shARID5B‐THP‐1 cells. (H) Schematic representation of the mutated sequences of potential ARID5B‐binding sites on the LINC01128 promoter; luciferase activity after transfection with a reporter containing wild‐type (WT‐LINC01128) or mutant LINC01128 (Mut‐LINC01128) promoter constructs in 293T cells. (I) RNA fluorescence in situ hybridisation (FISH) assay showing the subcellular localisation of LINC01128 in THP‐1 cells; U6 was used as a nuclear localisation control; green, LINC01128; red, U6; blue, DAPI. (J) Nuclear fractionation and RT‐qPCR analysis of LINC01128 expression in the nucleus and cytoplasm. Data information: error bars represent the mean ± SD of at least three independent experiments. ns, not significant; ^*^ *p* < .05; ^**^ *p* < .01; ^***^ *p* < .001. β2GPI, beta2‐glycoprotein I.

ARID5B‐bound regions are predominantly associated with active transcription,^39^ and lncRNA dysfunction has been shown to lead to autoimmune disorders and may contribute to APS.^6^ Therefore, anti‐ARID5B CUT&Tag was performed using THP‐1 cells to screen downstream lncRNAs involved in APS pathogenesis (Supporting Information S4). The results validated eight lncRNAs located within upstream 5 kb of the ARID5B's TSS (Figure 2A), and their transcription might be regulated by ARID5B. Among the eight potential lncRNAs, the binding sites of only ARID5B at the *LINC01128* promoter were predicted by the hTFtarget (Figure 2B). ARID5B did not influence the expression of other seven lncRNAs (Figure S1), thus, it was selected for further experiments.

To elucidate the underlying mechanisms of ARID5B in LINC01128 upregulation, we suppressed ARID5B levels using two specific shRNAs and increased its expression by transfecting with ARID5B overexpressing lentivirus (Figure 2C,D). Overexpression of ARID5B caused an increase in LINC01128 expression, whereas ARID5B knockdown dramatically downregulated LINC01128 and repressed IC‐induced LINC01128 expression in both THP‐1 cells and monocytes (Figure 2E,F). In addition, ARID5B depletion caused a 3.03‐fold reduction in the binding of ARID5B to the *LINC01128* promoter (Figure 2G). Considering that the two binding sites of ARID5B overlapped at the *LINC01128* promoter, one binding region was mutated (Figure 2H). The luciferase reporter assay further demonstrated that the mutated region significantly reduced the luciferase reporter activity of the *LINC01128* promoter (Figure 2H). These results suggested that ARID5B transcriptionally regulated LINC01128 expression in APS by activating its promoter.

Given that the function of LINC01128 is related to its subcellular localisation, we determined the subcellular distribution of LINC01128 using FISH and subcellular fractionation assays. The results demonstrated that LINC01128 was mostly expressed in the nucleus (Figure 2I,J).

### 3.3 LINC01128 regulated both pyroptosis and apoptosis in APS

**FIGURE 3:**
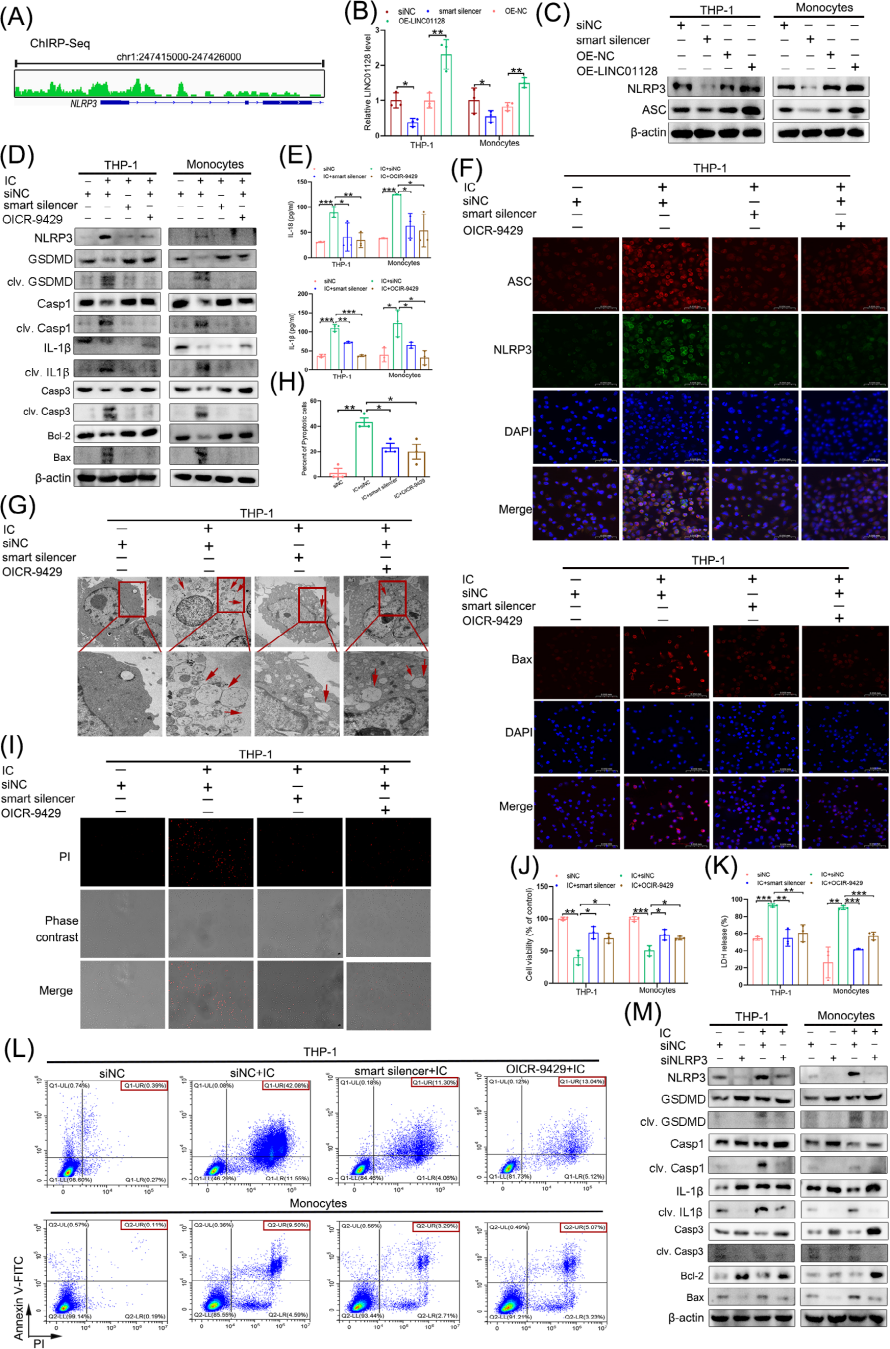
LINC01128 silencing repressed the key molecules involved in pyroptosis and apoptosis in antiphospholipid syndrome (APS). (A) Chromatin isolation by RNA purification (ChIRP) assay detected the enrichment of Nod‐like receptor family pyrin domain‐containing 3 (NLRP3) in the LINC01128 probe group in THP‐1 cells. (B) RT‐PCR analysis of LINC01128 expression after transfection with a smart silencer or overexpression plasmid in THP‐1 cells and monocytes. (C) Western blotting analysis of NLRP3 and apoptosis‐associated speck‐like protein (ASC) expression in LINC01128‐knockdown and LINC01128‐overexpressing THP‐1 cells and monocytes. (D) Western blotting revealed the expression of pyroptosis‐ and apoptosis‐related proteins in the small interfering negative control (siNC), siNC+IC, smart silencer‐LINC01128+IC and siNC+OICR‐9429+IC groups. (E) ELISA to detect the secretion of interleukin (IL)‐18 and IL‐1β in the siNC, siNC+IC, smart silencer‐LINC01128+IC and siNC+OICR‐9429+IC groups. (F) Immunofluorescence to detect the expression of NLRP3, ASC and Bax in the siNC, siNC+IC, smart silencer‐LINC01128+IC and siNC+OICR‐9429+IC groups in THP‐1 cells; original magnification, 40×. (G) Representative transmission electron microscopy (TEM) images and (H) the percentage of pyroptotic THP‐1 cells. Red arrows indicate the ballooned cell membrane or the large bubbles in the plasma. Scale bar, 2 μm. (I) Propidium iodide (PI) staining of THP‐1 cells in the siNC, siNC+IC, smart silencer‐LINC01128+IC and siNC+OICR‐9429+IC groups; original magnification, 20×. (J) Cell Counting Kit‐8 (CCK‐8) assay to assess the cell viability in the siNC, siNC+IC, smart silencer‐LINC01128+IC and siNC+OICR‐9429+IC groups in both THP‐1 cells and monocytes. (K) Release of lactate dehydrogenase (LDH) from THP‐1 cells and monocytes in siNC, siNC+IC, smart silencer‐LINC01128+IC and siNC+OICR‐9429+IC groups. (L) Flow cytometry analysis of THP‐1 cells and monocytes stained with Annexin V‐FITC and propidium iodide (PI); the percentage of double‐positive cells indicates pyroptotic cells labelled in red. (M) Western blotting to measure the levels of pyroptosis‐ and apoptosis‐related proteins in the siNC, siNLRP3, siNC+IC and siNLRP3+IC groups in both THP‐1 cells and monocytes. Data information: error bars represent the mean ± SD of at least three independent experiments. ^*^ *p* < .05; ^**^ *p* < .01; ^***^ *p* < .001. IC, β2GPI/anti‐β2GPI immune complex; β2GPI, beta2‐glycoprotein I.

To explore the downstream pathways of LINC01128, we performed the ChIRP assay with biotinylated oligo probes. The results indicated that LINC01128 physiologically interacted with the promoter sequence of NLRP3 (Figure 3A and Supporting Information 5). The active NLRP3 inflammasome not only stimulates pyroptosis but also apoptosis,^40^, ^41^ thereby participating in the pathogenesis of autoimmune diseases by inducing inflammation of monocytes.^42^ To confirm the effect of LINC01128 on NLRP3 expression, we depleted LINC01128 using a specific smart silencer and upregulated its transcription using a LINC01128 overexpressing plasmid (Figure 3B). LINC01128 positively regulated the expression of NLRP3 and apoptosis‐associated speck‐like protein (ASC) (Figure 3C). Therefore, we explored whether LINC01128 participated in pyroptosis and apoptosis in APS by regulating NLRP3 expression.

Next, we exposed THP‐1 cells and monocytes to IC and investigated whether IC treatment caused pyroptosis and apoptosis in THP‐1 cells and monocytes. The results verified that the expression of NLRP3, clv. GSDMD, clv. Casp1, clv. IL‐1β and the Bax/Bcl‐2, clv. Casp3 were upregulated in the IC group, whereas their expression was downregulated in the smart silencer‐LINC01128+IC and siNC+OICR‐9429+IC groups (Figure 3D). The secretory levels of IL‐18 and IL‐1β were also augmented in the siNC+IC group and suppressed in the smart silencer‐LINC01128+IC and siNC+OICR‐9429+IC groups (Figure 3E). Immunofluorescence showed that IC stimulation markedly upregulated the protein levels of Bax, ASC and NLRP3, whereas OICR‐9429 treatment or LINC01128 depletion decreased their levels (Figure 3F). TEM indicated that IC‐treated THP‐1 cells were more prone to pore information, cell swelling, rupture and typical morphological features of cell pyroptosis (Figure 3G). Compare to the siNC+IC group, the percentage of pyroptotic cells in the smart silencer‐LINC01128+IC and siNC+OICR‐9429+IC groups was lower (Figure 3H). Consistent with this observation, IC exposure effectively decreased the viability of monocytes and THP‐1 cells compared to that in the smart silencer‐LINC01128+IC and siNC+OICR‐9429+IC groups (Figure 3J). PI is permeable into cells upon pyroptosis, inducing the breakage of the plasma membrane. IC treatment increased membrane disruption and PI fluorescence, and OICR‐9429 or LINC01128 knockdown attenuated PI fluorescence (Figure 3I). Cytosolic components were secreted from the ruptured cytoplasmic membrane. Therefore, LDH levels were examined as a cytotoxic indicator of pyroptotic cells. IC stimulation substantially enhanced the production of LDH, whereas its production was suppressed in the smart silencer‐LINC01128+IC and siNC+OICR‐9429+IC groups (Figure 3K). Flow cytometry analysis indicating double positive rate of PI and Annexin V revealed IC‐induced pyroptotic cell death, and the double positive rate of PI and Annexin V was decreased in the smart silencer‐LINC01128+IC and siNC+OICR‐9429+IC groups (Figure 3L). These data suggested that OICR‐9429 treatment or LINC01128 knockdown might specifically mitigate IC‐induced pyroptotic cell death in an in vitro model mimicked APS.

For further clarifying whether the impact of LINC01128 on pyroptosis and apoptosis in APS was NLRP3 dependent, NLRP3 was specifically silenced in THP‐1 cells and monocytes by siRNA transfection. The data validated that NLRP3 deletion reduced the protein levels of clv. GSDMD, clv. Casp1, clv. IL‐1β, the Bax/Bcl2 ratio and clv. Casp3 in the siNLRP3 and siNLRP3+IC groups compared to those in the siNC and siNC+IC groups (Figure 3M). Collectively, LINC01128 positively regulated both pyroptosis and apoptosis in APS via NLRP3 signalling.

### 3.4 LINC01128 promoted the formation of the BTF3/STAT3 complex

**FIGURE 4:**
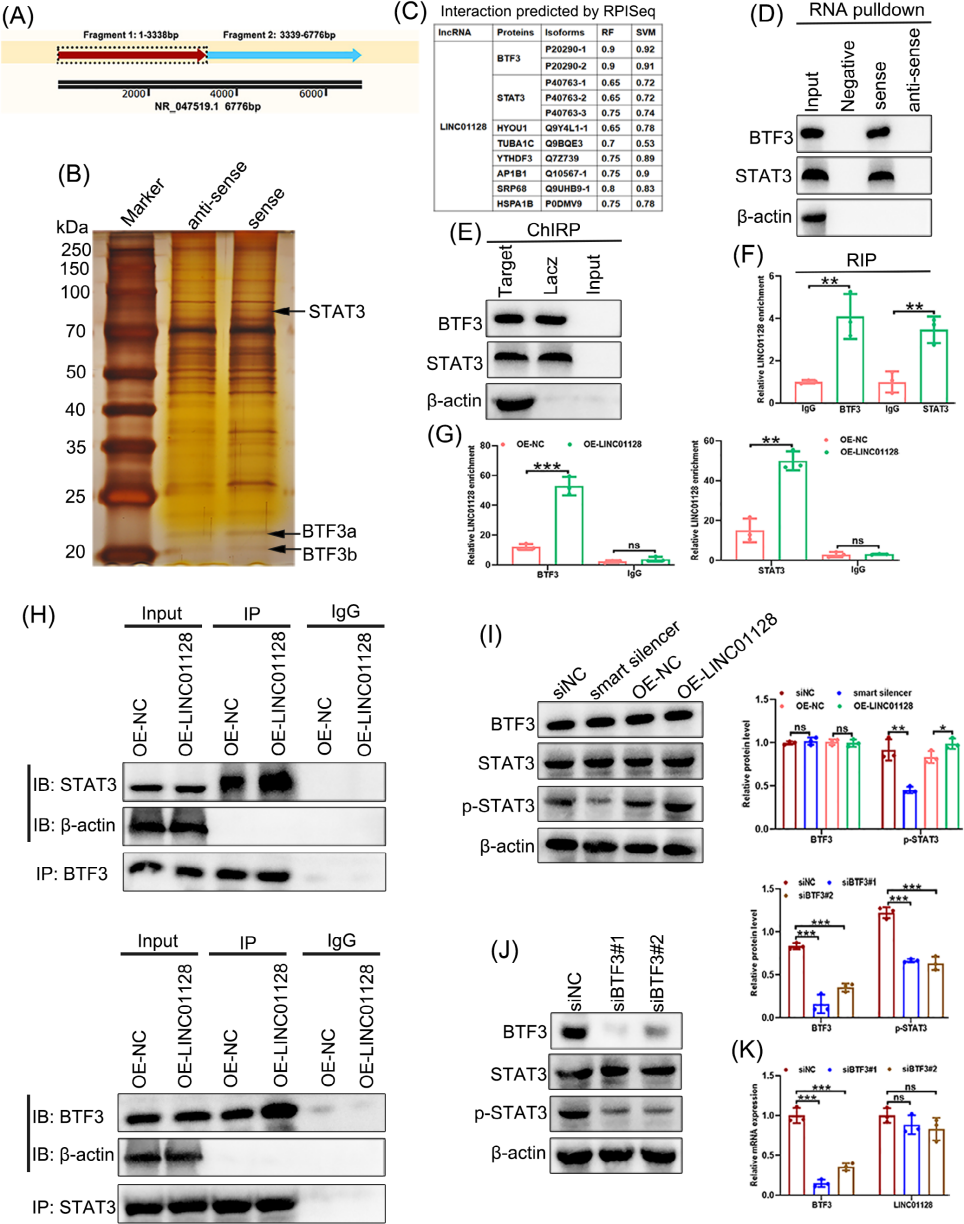
LINC01128 promoted the formation of the BTF3/STAT3 complex. (A) Schematic representation of the two biotinylated fragments of LINC01128. (B) RNA pull‐down assay using LINC01128 sense and antisense RNA in THP‐1 cells, followed by silver staining. (C) The interaction score of LINC01128 with BTF3, STAT3 and other proteins was predicted using http://pridb.gdcb.iastate.edu/RPISeq/about.php (RNA‐Protein Interaction Prediction, RPISeq). (D) Western blotting detected the interaction of LINC01128 with BTF3 and STAT3 using the extract of the RNA pull‐down assay. (E) Western blotting to measure the interaction of LINC01128 with BTF3 and STAT3 using the extract of the chromatin isolation by RNA purification (ChIRP) assay. (F) RNA immunoprecipitation (RIP)‐qPCR analysis of LINC01128 with anti‐BTF3 or anti‐STAT3 antibody in THP‐1 cells. (G) RIP‐qPCR analysis of LINC01128 with anti‐BTF3 or anti‐STAT3 antibody in OE‐NC and OE‐LINC01128 THP‐1 cells. (H) Co‐immunoprecipitation (Co‐IP) analysis of OE‐NC and OE‐LINC01128 THP‐1 cells using anti‐BTF3 or anti‐STAT3 antibody; western blotting to verify the Co‐IP products using anti‐STAT3 or anti‐BTF3 antibody. (I) Western blotting to detect the expression of BTF3 and p‐STAT3/STAT3 in THP‐1 cells after knockdown or overexpression of LINC01128. (J) Western blotting to identify the expression of BTF3 and p‐STAT3/STAT3 in THP‐1 cells after the knockdown of BTF3. (K) Real‐time quantitative PCR (RT‐qPCR) to detect the expression of BTF3 and LINC01128 in THP‐1 cells after the knockdown of BTF3. Data information: error bars represent the mean ± SD of at least three independent experiments. ns, not significant; ^*^ *p* < .05; ^**^ *p* < .01; ^***^ *p* < .001.

The function of lncRNAs is correlated with the formation of protein complexes. Therefore, we hypothesised that LINC01128 might participate in NLRP3‐mediated pyroptosis and apoptosis by binding to proteins. Given that the full‐length sequence of LINC01128 is excessively long, we truncated LINC01128 into two biotinylated fragments (Figure 4A). Then, we performed RNA pull‐down and mass spectrometry assays to capture RNA‐binding proteins (Figure 4B and Supporting Information 6 and 7). The detected RNA‐binding proteins included HSPA1B, TUBA1C, YTHDF3, SRP68, AP1B1, HYOU1, BTF3 and STAT3. The binding of LINC01128 to these proteins was successfully predicted using RPISeq (Figure 4C). However, the relationship between HSPA1B, TUBA1C, YTHDF3, SRP68, AP1B1, HYOU1 and pyroptosis has not been reported. BTF3, a basic transcription factor, has been shown to promote STAT3 phosphorylation, and activated STAT3 signalling potentially modulates NLRP3‐mediated pyroptosis and associated inflammation.^43^, ^44^ Thus, the transcription factors BTF3 and STAT3 were of particular interest.

The RNA pull‐down and western blotting results showed that BTF3 and STAT3 were bound by the biotinylated LINC01128 fragment 1 (Figure 4D). Moreover, the ChIRP and western blotting results confirmed that LINC01128 is bound to both BTF3 and STAT3 (Figure 4E). Following the above results, a RIP assay using anti‐BTF3 or anti‐STAT3 antibodies revealed that LINC01128 strongly interacted with BTF3 and STAT3 compared to IgG (Figure 4F), and overexpression of LINC01128 increased LINC01128 enrichment in THP‐1 cells (Figure 4G).

LINC01128 overexpression promoted the effective interacting of BTF3 with STAT3 (Figure 4H) to positively regulate STAT3 phosphorylation (Figure 4I). However, LINC01128 depletion or overexpression had no impact on the levels of BTF3 or STAT3 (Figure 4I). To verify the effect of BTF3 on STAT3 phosphorylation, two siRNAs were utilised for the depletion of BTF3, and the data validated that BTF3 knockdown inhibited STAT3 phosphorylation (Figure 4J) and did not influence LINC01128 expression (Figure 4K). Collectively, LINC01128 promoted the formation of the BTF3/STAT3 complex and enhanced STAT3 phosphorylation.

### 3.5 LINC01128 promoted p‐STAT3‐mediated pyroptosis and apoptosis pathways

**FIGURE 5:**
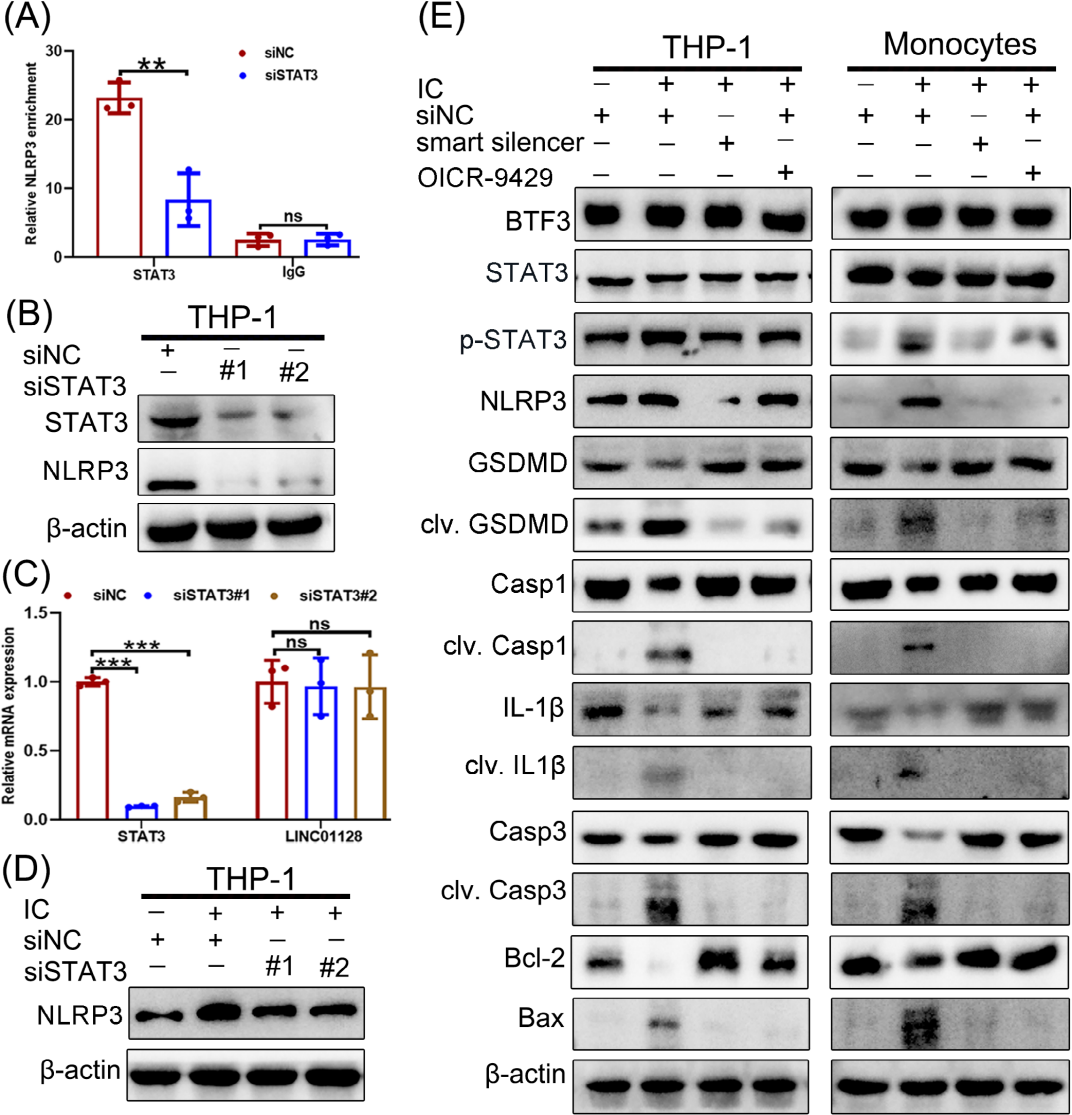
LINC01128 promoted p‐STAT3‐mediated pyroptosis and apoptosis pathways. (A) Chromatin immunoprecipitation (ChIP)‐qPCR to detect the enrichment of p‐STAT3 at the *NLRP* promoter using the anti‐p‐STAT3 antibody in small interfering negative control (siNC)‐ and siSTAT3‐THP‐1 cells. (B) Western blotting to detect the expression of STAT3 and Nod‐like receptor family pyrin domain‐containing 3 (NLRP3) in THP‐1 cells after the knockdown of STAT3. (C) Real‐time quantitative PCR (RT‐qPCR) to detect the expression of STAT3 and LINC01128 in THP‐1 cells after the knockdown of STAT3. (D) Western blotting to identify the expression of NLRP3 in the siNC, siNC+IC, siSTAT3#1+IC and siSTAT3#2+IC groups. (E) Western blotting to identify the expression of BTF3 and p‐STAT3/STAT3, and the levels of pyroptosis‐ and apoptosis‐related proteins in THP‐1 cells and monocytes in the siNC, siNC+IC, smart silencer‐LINC01128+IC and siNC+OICR‐9429+IC groups. Data information: error bars represent the mean ± SD of at least three independent experiments. ns, not significant; ^**^ *p* < .01; ^***^ *p* < .001. IC, β2GPI/anti‐β2GPI immune complex; β2GPI, beta2‐glycoprotein I.

Several reports have demonstrated that p‐STAT3 directly upregulates NLRP3 expression by enhancing histone H3 and H4 acetylation at its promoter.^44^, ^45^ To further validate the regulation of p‐STAT3 in mediating NLRP3 in APS, we performed ChIP‐qPCR and found that p‐STAT3 bound to the *NLRP3* promoter in THP‐1 cells (Figure 5A). Then, we depleted STAT3 using two specific siRNAs (Figure 5B,C). STAT3 knockdown significantly decreased the binding of p‐STAT3 to the *NLRP3* promoter (Figure 5A) and reduced NLRP3 expression (Figure 5B) and not LINC01128 transcription (Figure 5C). STAT3 depletion also downregulated IC‐induced NLRP3 expression (Figure 5D).

After confirming that p‐STAT3 modulated NLRP3 expression by binding to its promoter, we further examined whether LINC01128 affected the p‐STAT3/STAT3 ratio in the in vitro model mimicked APS. The expression of p‐STAT3/STAT3, NLRP3, clv. GSDMD, clv. Casp1, clv. IL‐1β, clv. Casp3 and Bax/Bcl‐2 was markedly increased in the siNC+IC groups of THP‐1 cells and monocytes. However, the expression of these biomarkers was repressed in the smart silencer‐LINC01128+IC and siNC+OICR‐9429+IC groups, and the expression of BTF3 was not significantly different (Figure 5E). Therefore, LINC01128 modulated NLRP3 expression by promoting STAT3 phosphorylation, which further triggered pyroptosis and apoptosis in APS.

### 3.6 ARID5B‐mediated LINC01128 promoted pyroptosis and apoptosis via the p‐STAT3 axis

**FIGURE 6:**
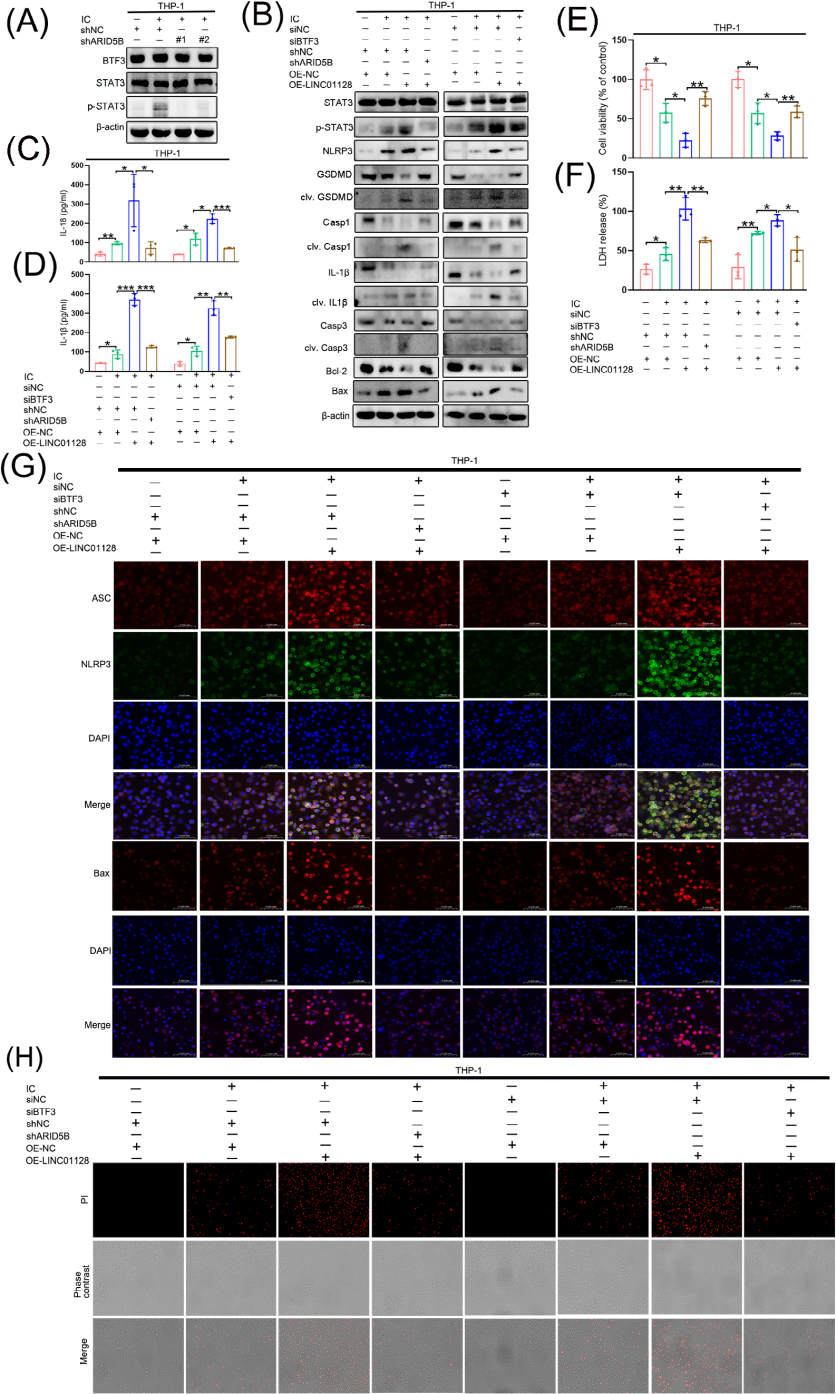
ARID5B or BTF3 knockdown interfered with LINC01128‐mediated pyroptosis and apoptosis via the p‐STAT3 pathway. (A) Western blotting to identify the expression of BTF3 and p‐STAT3/STAT3 after ARID5B knockdown in an in vitro THP‐1 cell model of antiphospholipid syndrome (APS). (B) Western blotting showed the levels of p‐STAT3/STAT3, and pyroptosis‐ and apoptosis‐related proteins after LINC01128 overexpression and ARID5B or BTF3 knockdown in an in vitro THP‐1 cell model of APS. (C and D) ELISA showed the secretory levels of interleukin (IL)‐18 and IL‐1β, respectively, after LINC01128 overexpression and ARID5B or BTF3 knockdown in an in vitro THP‐1 cell model of APS. (E) CCK‐8 assay and (F) lactate dehydrogenase (LDH) assay after LINC01128 overexpression and ARID5B or BTF3 knockdown in an in vitro THP‐1 cell model of APS. (G) Immunofluorescence to detect the expression of Nod‐like receptor family pyrin domain‐containing 3 (NLRP3), ASC and Bax after LINC01128 overexpression and ARID5B or BTF3 knockdown in an in vitro THP‐1 cell model of APS. (H) Propidium iodide (PI) staining after LINC01128 overexpression and ARID5B or BTF3 knockdown in an in vitro THP‐1 cell model of APS; original magnification, 20×. Data information: error bars represent the mean ± SD of at least three independent experiments. ns, not significant; ^*^ *p* < .05; ^**^ *p* < .01; ^***^ *p* < .001. IC, β2GPI/anti‐β2GPI immune complex; β2GPI, beta2‐glycoprotein I.

Furthermore, this study elucidated whether ARID5B and BTF3 stimulated the NLRP3 pathway in THP‐1 cells. The results showed that shRNA‐mediated ARID5B depletion did not affect the level of BTF3. However, the p‐STAT3/STAT3 levels decreased in the shARID5B#1+IC and shARID5B#2+IC groups compared to those in the shNC+IC group (Figure 6A). To elucidate the effects of ARID5B and BTF3 on LINC01128‐mediated pyroptosis and apoptosis, we performed rescue experiments to validate the regulatory mechanisms of the ARID5B/LINC01128/BTF3 axis. These data indicated that the levels of p‐STAT3‐mediated pyroptosis‐ and apoptosis‐related molecules and the secretory levels of IL‐18 and IL‐1β were augmented in the shNC+OE‐LINC01128+IC group compared to those in the shNC+OE‐NC+IC group (Figures 6B–D and S3A,B). However, co‐transfection with ARID5B knockdown and LINC01128 overexpression constructs had a compensatory effect on the activity of pyroptosis and apoptosis pathways (Figures 6B and S3A,B), downregulated the secretory IL‐18 and IL‐β (Figure 6C,D), and partially reduced the augmented fluorescence of NLRP3, ASC and Bax (Figures 6G and S3C). Similar results were obtained after co‐transfection with BTF3 knockdown and LINC01128 overexpression constructs (Figures 6B–D,G and S3A–C). In addition, the CCK8 assay (Figures 6E and S3D), LDH assay (Figures 6F and S3E) and PI staining (Figures 6H and S3F) indicated that knockdown of ARID5B and BTF3 partially rescued LINC01128‐induced pyroptosis and apoptosis of THP‐1 cells. Therefore, ARID5B‐mediated LINC01128 induced canonical pyroptosis and apoptosis in APS via activation of the BTF3/STAT3 pathway.

### 3.7 The activation of ARID5B/LINC01128/BTF3/STAT3 signalling in mice with APS

**FIGURE 7:**
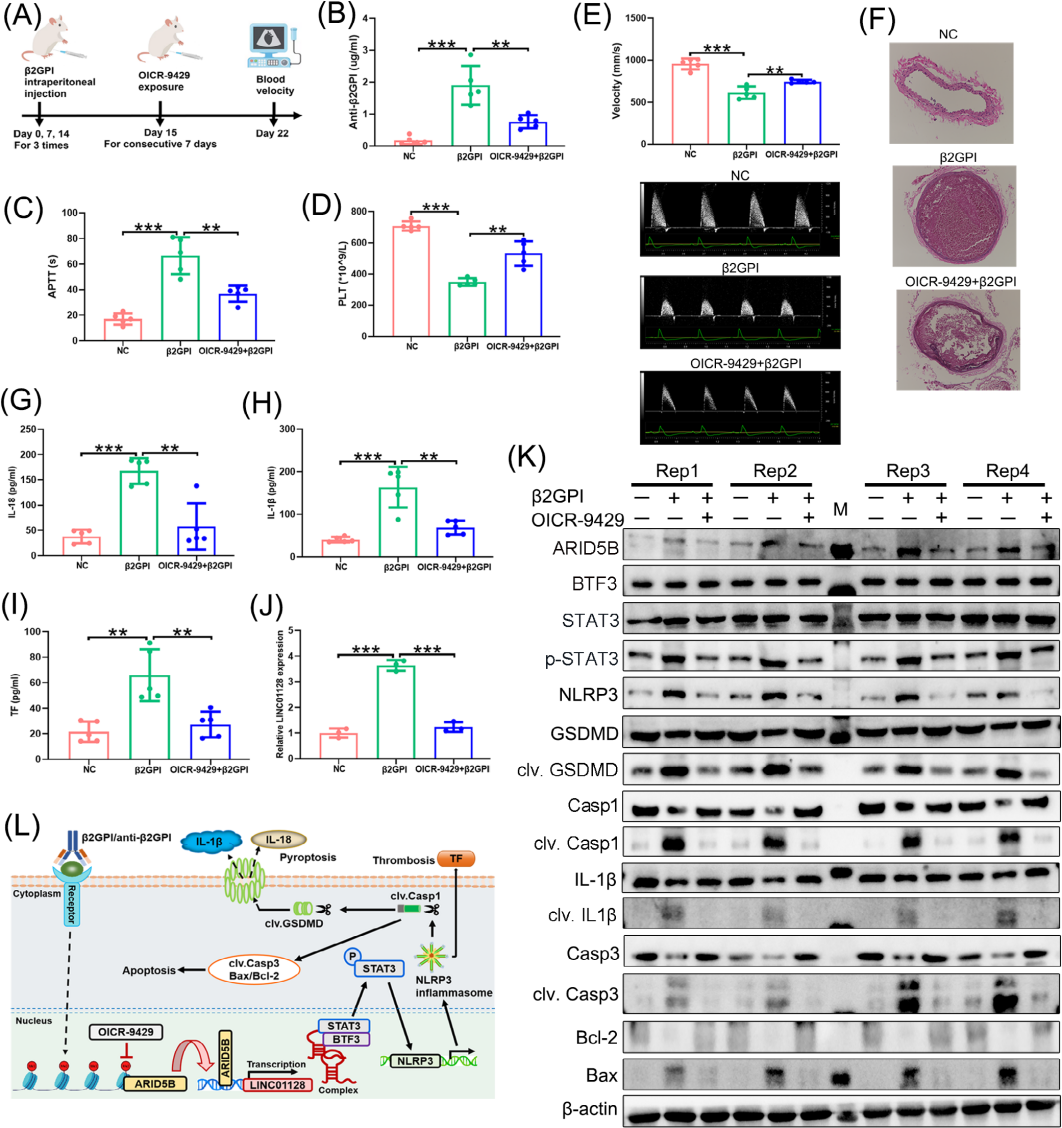
The activation of ARID5B/LINC01128/BTF3/STAT3 signalling in mice with antiphospholipid syndrome (APS). (A) Workflow of beta2‐glycoprotein I (β2GPI) intraperitoneal injection and OICR‐9429 exposure, β2GPI was injected once a week for 3 weeks to generate mice with vascular APS in vivo (number of mice in each group = 5). (B) Anti‐β2GPI levels, (C) activated partial thromboplastin time (APTT) and (D) platelet count (PLT) were detected in the negative control (NC), β2GPI and OICR‐9429+β2GPI groups. (E) The blood velocity of the ascending aorta was tested using Doppler ultrasound in the three groups of mice. (F) The thrombus size of the carotid artery was tested by haematoxylin–eosin (HE) staining in the three groups of mice; original magnification, 100×. (G–I) ELISA displaying the serum levels of interleukin (IL)‐18, IL‐1β and tissue factor (TF), respectively. (J) Real‐time quantitative PCR (RT‐qPCR) to detect the expression of LINC01128 in the three groups of mice. (K) Western blotting indicated the expression of ARID5B, BTF3 and p‐STAT3/STAT3, the activity of pyroptosis and apoptosis pathways in the three groups of mice. (L) Schematic diagram illustrating the potential mechanism of ARID5B‐mediated LINC01128 in p‐STAT3‐induced pyroptosis and apoptosis activation in APS. Data information: error bars represent the mean ± SD of at least three independent experiments. M, marker; ns, not significant; ^**^ *p* < .01; ^***^ *p* < .001.

To validate the in vitro findings in vivo, our study generated a mouse model mimicked APS by intraperitoneal β2GPI injection thrice (Figure 7A).^33^, ^34^, ^35^ Then, one group of mice with APS were administered with OICR‐9429 for 7 consecutive days (Figure 7A). Compared to that in the NC and OICR‐9429+β2GPI groups, β2GPI group mimicked APS had higher anti‐β2GPI levels (Figure 7B), longer APTT (Figure 7C), fewer PLT (Figure 7D), slower blood velocity of the ascending aorta (Figure 7E) and a larger thrombus size of the carotid artery (Figure 7F), indicating that OICR‐9429 exerted an opposite effect. Furthermore, the secretory IL‐18, IL‐1β and TF were dramatically augmented in the β2GPI group, and their release was blocked in the NC and OICR‐9429+β2GPI groups (Figure 7G–I). Bone marrow‐derived monocytes were extracted from mice femurs to detect the expression of relevant molecules. Results indicated the upregulated ARID5B, LINC01128 and p‐STAT3/STAT3 as well as active downstream pyroptotic and apoptotic pathways in the β2GPI group, and their activation was inhibited in the NC and OICR‐9429+β2GPI groups (Figure 7J,K). The difference of BTF3 expression between the three groups was no significance. These results indicated that ARID5B‐mediated LINC01128 regulated pyroptosis and apoptosis via the BTF3/p‐STAT3 axis, exacerbating inflammation and thrombosis in mice with APS, and that OICR‐9429 could relieve APS progression by blocking the above pathways (Figure 7L).

### 3.8 The correlation of ARID5B/LINC01128 with aPLs in patients with PAPS

**FIGURE 8:**
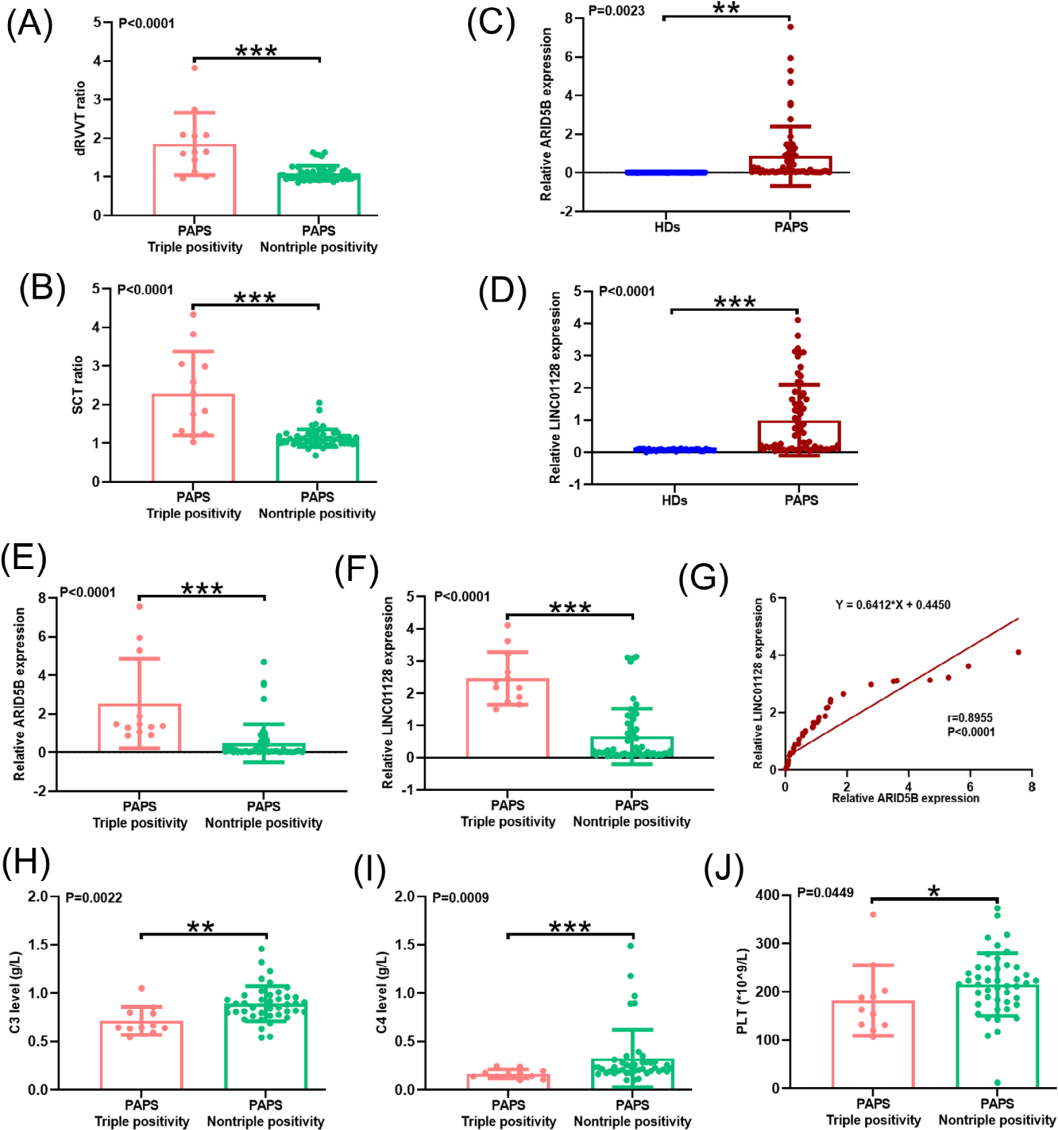
The correlation of ARID5B/LINC01128 with antiphospholipid antibodies (aPLs) in patients with primary antiphospholipid syndrome (PAPS). (A) dilute Russell's viper venom time (dRVVT) ratio and (B) silica clotting time (SCT) ratio of triple‐positive (*N* = 12) and non‐triple‐positive (*N* = 52) patients with PAPS. (C) Relative ARID5B and (D) LINC01128 expression in patients with PAPS (*N* = 64) and healthy donors (HDs) (*N* = 32). (E) Relative ARID5B and (F) LINC01128 expression in triple‐positive (*N* = 12) and non‐triple‐positive (*N* = 52) patients with PAPS. (G) Correlation analysis between the expression of ARID5B and LINC01128. (H) C3, (I) C4 and (J) platelet count (PLT) levels in triple‐positive (*N* = 11) and non‐triple‐positive (*N* = 42, 43, 45) patients with PAPS. Data information: error bars represent the mean ± SD of at least three independent experiments. ^*^ *p* < .05; ^**^ *p* < .01; ^***^ *p* < .001.

In the clinical setting, we included 64 patients with PAPS and 32 HDs. The patients’ demographic characteristics and clinical findings are outlined (Table S1 and Supporting Information 3). Twelve patients with PAPS had triple positivity for aPLs. Nineteen patients had double positivity for aPLs: 26.32% (5/19) double positivity for aCL and lupus anticoagulant (LAC), 31.58% (6/19) double positivity for anti‐β2GPI and LAC, and 42.11% (8/19) double positivity for aCL plus anti‐β2GPI. Thirty‐three patients with PAPS had single positivity for aPLs: 45.45% (15/33) single positivity for LAC or aCL and 9.09% (3/33) single positivity for anti‐β2GPI. Patients with triple positivity represented a higher dRVVT or SCT ratio than those with non‐triple positivity (Figure 8A,B and Table S1).

To explore the clinical value of ARID5B/LINC01128 in APS progression, we extracted peripheral blood monocytes from patients with PAPS and HDs and examined the mRNA expression of ARID5B and LINC01128. Patients with PAPS had dramatically increased expression of ARID5B and LINC01128 compared to that in HDs (Figure 8C,D), and triple‐positive patients had the higher expression of ARID5B and LINC01128 compared to non‐triple‐positive patients (Figure 8E,F). Furthermore, there was a positive correlation between ARID5B and LINC01128 expression in patients with PAPS (Figure 8G; *r* = .8955, *p* < .0001).

Complement C3 and C4 levels and platelets affect APS pathogenesis. Next, we assessed the levels of C3, C4 and PLT in patients with PAPS. Only 53, 54 and 56 patients had detectable levels of C3, C4 and PLT, respectively. Results indicated that triple‐positive patients had the significantly lower C3, C4 and PLT levels compared to non‐triple‐positive patients (Figure 8H–J and Table S1). Nevertheless, the positivity of aPLs did not correlate with the history of thrombosis or APOs (Table S1). These findings suggested that ARID5B and LINC01128 were synergistically increased in patients with PAPS, which is related to the increased positivity of aPLs. Moreover, aPLs might have an impact on the levels of C3, C4 and PLT.

## 4 DISCUSSION

To date, the mechanisms underlying APS have not been completely elucidated. APS is heterogeneous based on molecular aberrations and clinical features, thus, the investigation of the aetiology and the treatment methods is complicated.^46^ Additionally, the APS's course is rather long and difficult to cure, resulting in accumulative alterations in molecular function and cell structure. H3K4me3 is a classical epigenetic mediator that acknowledged as an excellent‐tuned pattern for orchestrating genes.^47^

Our data confirmed that H3K4me3‐mediated ARID5B participated in APS pathogenesis. ARID5B, termed modulator recognition factor‐2, is a transcription factor with a DNA‐binding motif.^48^ Moreover, ARID5B is upregulated in an in vitro monocyte model mimicked APS and in monocytes from patients with cardiovascular disease,^5^, ^49^ and is critical for the inflammatory response in lipopolysaccharide (LPS)‐stimulated macrophages. In our experiments, the transcriptional regulation of ARID5B displayed an essential epigenetic component in response to stimulation and was involved in orchestrating LINC01128 expression.

lncRNAs are a typical category of regulatory molecules and have been previously reported to influence autoimmune diseases through gene regulatory networks. The hsa‐miR‐21‐5p/PTX3 network modulated by LINC01128 regulates immune response and relevant inflammation in systemic sclerosis.^50^, ^51^ In this study, we found that LINC01128 enhanced the formation of the BTF3/STAT3 complex, thus facilitating the activation of the p‐STAT3 pathway. Overexpression of LINC01128 did not affect the expression of BTF3 and STAT3. However, it enhanced the binding of STAT3 to BTF3 and the phosphorylation of STAT3, demonstrating that the binding efficiency positively modulated the pathway. One the other side, BTF3 has been reported to regulate the level of p‐STAT3/STAT3 but does not change the total STAT3.^43^, ^52^ Consistently, our findings showed that BTF3 interference downregulated p‐STAT3/STAT3 levels.

STAT3 is a crucial transcriptional regulator of pyroptosis, apoptosis and related inflammation. Moreover, pyroptosis and apoptosis are two vital pathways that are implicated in APS pathogenesis.^17^, ^53^ A previous study confirmed that active STAT3 upregulates NLRP3 via directly enhancing histone H3 and H4 acetylation at the *NLRP3* promoter in LPS‐treated macrophages.^45^ The upregulated NLRP3 triggered canonical pyroptosis and apoptosis, and also induced the release of proinflammatory cytokines in autoimmune diseases, implying its promising therapeutic potential.^54^, ^55^ In line with the above observations, our results revealed that p‐STAT3 positively regulated NLRP3 expression at its promoter and triggered related pyroptosis and apoptosis. These results demonstrated that LINC01128 induced pyroptosis and apoptosis in APS via the p‐STAT3 pathway. Depletion of ARID5B or BTF3 partially suppressed pyroptosis and apoptosis induced by LINC01128 overexpression.

Aligning with the in vitro findings, the expression of ARID5B, LINC01128 and p‐STAT3/STAT3 was upregulated, and NLRP3 inflammasome‐induced pyroptosis and apoptosis pathways were activated in mice with APS, indicating that the ARID5B‐mediated LINC01128/BTF3/STAT3 axis had an impact on accelerating APS pathogenesis in vivo. Moreover, the levels of BTF3 and STAT3 did not significantly increase between the NC, β2GPI and OICR‐9429+β2GPI groups, further indicating that LINC01128 facilitated the formation of the BTF3/STAT3 protein complex. Previous findings have identified that inflammasome activation stimulated the release of TF from pyroptotic monocytes and macrophages, consequently contributing to arterial and venous thrombosis, uncovering an important link between inflammation and thrombosis.^56^, ^57^ Suppression of NLRP3 inflammasome components may provide prospective therapeutic targets for cardiovascular disease.^58^, ^59^ In this study, we detected an active NLRP3 inflammasome, increased TF secretion, and increased thrombus size in mice with APS. An increased thrombus size has been reported to be linked with reduced blood flow velocity.^60^ Thus, we concluded that the upregulated NLRP3 was responsible for the increased thrombus size, reduced blood velocity of the ascending aorta, and enhanced release of TF. However, the functional mechanisms of the NLRP3 inflammasome in thrombosis in APS must be explored in future experiments. OICR‐9429, the epigenetic inhibitor of H3K4me3, ameliorated the secretory IL‐18, IL‐1β and TF, demonstrating the inhibitive function for inflammation and thrombosis in mice with APS and a prospective area of investigation for APS therapy.

Finally, the levels of ARID5B and LINC01128 were assessed in patients with PAPS and HDs. ARID5B and LINC01128 were highly expressed in patients with PAPS; however, their role in the development of APS needs further investigation based on longitudinal data and clinical manifestations. ARID5B was confirmed to involve in the formation of inflammation and thrombosis in atherosclerosis.^49^, ^61^ Our study identified that ARID5B and regulated LINC01128 were higher in patients with PAPS with triple positivity, confirming that ARID5B/LINC01128 positively influenced the positive rate of aPLs. Nevertheless, no literatures reported how ARID5B/LINC01128 influenced the positive rate of aPLs, which is worth exploring and would be the focus in our next study. APLs, particularly LAC and aCL, contribute to thrombosis and thrombocytopenia in APS.^62^ In line with this, our data showed that patients with PAPS with triple positivity had fewer PLT compared to that in non‐triple‐positive patients. Complement levels are associated with the disease activity of APS.^63^, ^64^ Studies have shown that the levels of C3 and C4 are decreased in patients with APS with thrombotic events,^63^ and the reduced complement levels are associated with the increased risk of APOs.^64^ Although we observed that triple‐positive patients with PAPS had decreased C3 and C4 levels compared to that in non‐triple‐positive patients, the positivity of aPLs had no significant relationship with the history of thrombosis or APOs. The reason for this result may be that the majority of patients with PAPS who visit our hospital have a history of adverse pregnancy; therefore, more samples should be collected to confirm the relationship between aPLs positivity and the history of thrombosis and APOs.

## 5 CONCLUSION

In conclusion, this study identifies that IC enhance LINC01128 expression in an in vitro THP‐1 cell and monocyte model of APS via the epigenetic factor ARID5B. LINC01128 binds to the BTF3/STAT3 complex and enhances the activity of the p‐STAT3 pathway, ultimately inducing pyroptosis and apoptosis, and is associated with APS pathogenesis. The activation of ARID5B/LINC01128/p‐STAT3 in mice with APS triggers inflammation and thrombosis, aligning with the in vitro findings. Moreover, ARID5B expression is positively related with LINC01128 transcription in patients with PAPS. Overall, the findings described here suggest the pathogenic mechanisms of ARID5B/LINC01128 in APS and the potential of OICR‐9429 in APS therapy. However, the potential of ARID5B/LINC01128 as prognostic biomarkers for patients with APS needs to be further assessed.

## AUTHOR CONTRIBUTIONS

Yuan Tan and Liyan Cui contributed to the study conception and design. The first draft of the manuscript and Figures 1, 2, 3, 4, 5, 6 were prepared by Yuan Tan. Figures 7 and 8 were prepared by Zhongxin Li and Boxin Yang. The experiments were performed by Yuan Tan, Jiao Qiao and Shuo Yang. Data analysis was performed by Hongchao Liu. Clinical samples were collected by Qingchen Wang, Weimin Feng and Qi Liu. All authors commented on previous versions of the manuscript and read and approved the final manuscript.

## CONFLICT OF INTEREST STATEMENT

The authors declare they have no conflicts of interest.

## ETHICS STATEMENT

All experiments involving animals were approved by the Ethics Committee of Peking University Third Hospital (approval form: 060‐02). The studies involving human participants were approved by Ethics Committee of Peking University Third Hospital (approval form: 053‐01). Written informed consent for participation was not required for this study in accordance with the national legislation and the institutional requirements.

## Supporting information

Supporting information

Supporting information

Supporting information

Supporting information

## Data Availability

Raw sequencing data have been deposited at National Genomic Data Center (https://ngdc.cncb.ac.cn/gsa/) and will be accessed soon after publication (accession number: CRA013537). Other datasets generated during this study are included in this published article and its Supporting Information. Additional datasets analysed during the current study are available from the corresponding author on reasonable request.
