# Supplementary material for: ARID5B‐mediated LINC01128 epigenetically activated pyroptosis and apoptosis by promoting the formation of the BTF3/STAT3 complex in β2GPI/anti‐β2GPI‐treated monocytes
Source: Clin Transl Med. 2024 Jan 15;14(1):e1539. doi: 10.1002/ctm2.1539 (PMC10788880; doi:10.1002/ctm2.1539)
Supplement: Supplementary file 1 — Supporting information [file CTM2-14-e1539-s001.docx]

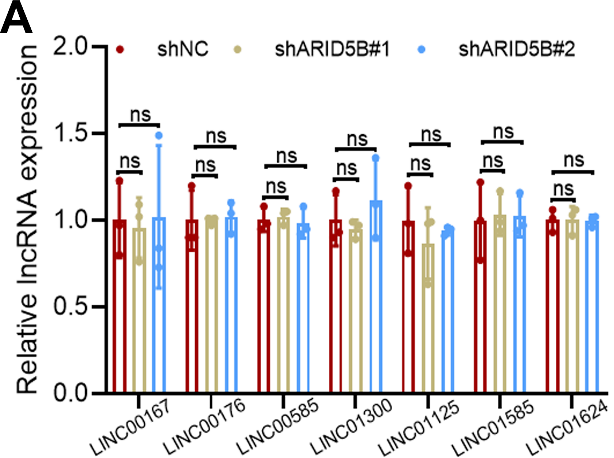


**Figure S1.** The RT-qPCR data of 7 lncRNAs. **A** The RT-qPCR data of LINC00167, LINC00176, LINC00585, LINC01300, LINC01585, and LINC01624 in shNC-, shARID5B#1-, and shARID5B#2-THP-1 cells. ns, not significant.


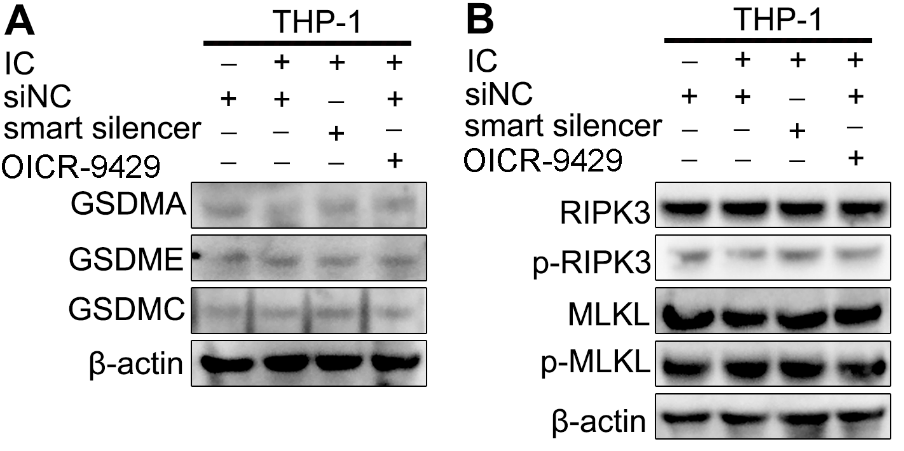


**Figure S2.** LINC01128 silencing had no impact on NLRP3-independent pyroptosis and necrosis in APS. **A** The expression of GSDMA, GSDME, and GSDMC in siNC, siNC+IC, smart silencer-LINC01128+IC, and siNC+OICR-9429+IC groups. **B** The expression of necrosis-associated molecules in siNC, siNC+IC, smart silencer-LINC01128+IC, and siNC+OICR-9429+IC groups.


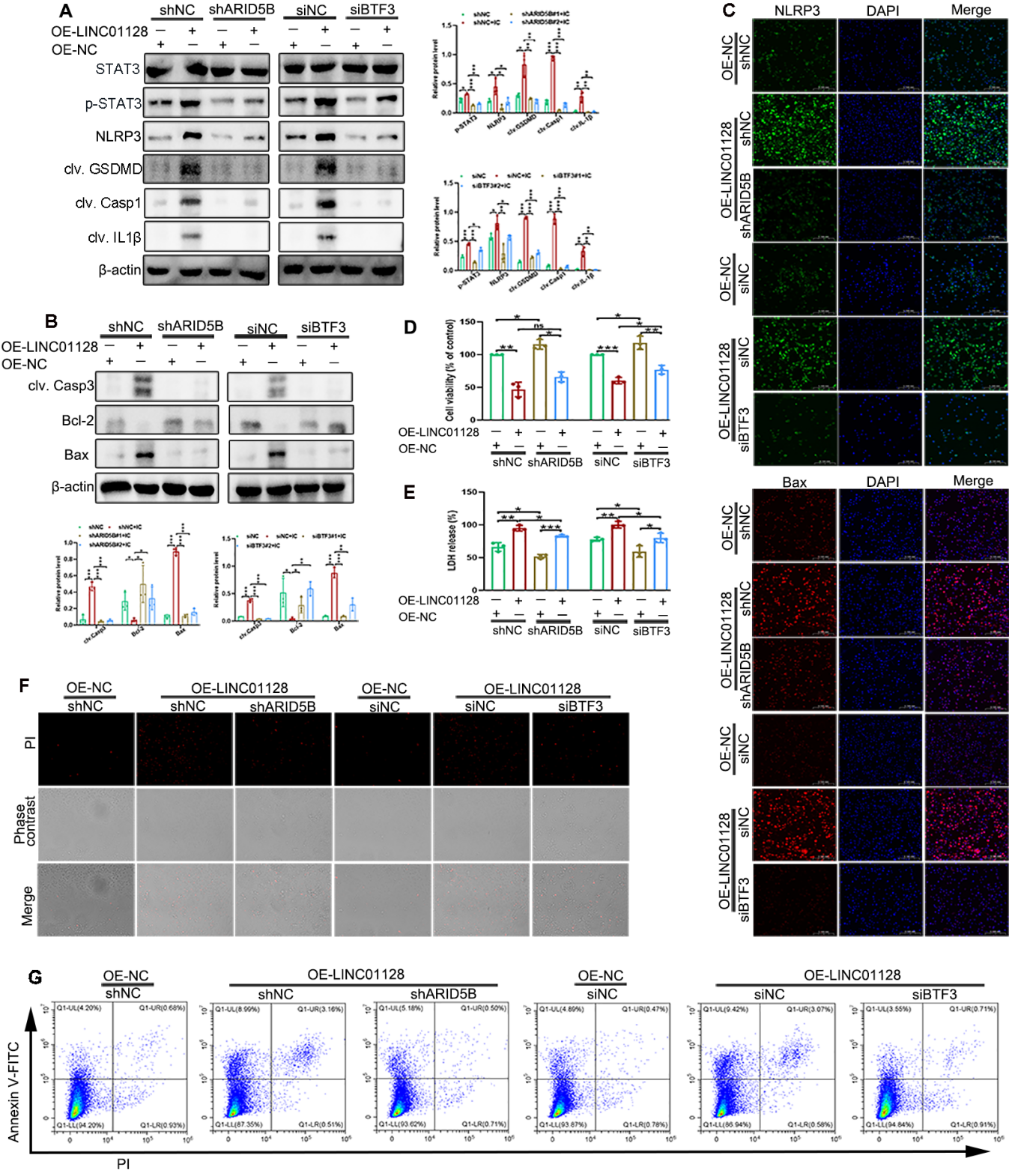


**Figure S3.** ARID5B or BTF3 knockdown interfered LINC01128-mediated pyroptosis and apoptosis via the p-STAT3 pathway. **A** Western blotting identified the level of p-STAT3/STAT3 and pyroptosis-related molecules in THP-1s after LINC01128 overexpression and ARID5B or BTF3 knockdown. **B** Western blot showing the activity of apoptosis pathway in THP-1s after LINC01128 overexpression and ARID5B or BTF3 knockdown. **C** Immunofluorescence detection of NLRP3 and Bax in THP-1 cells after LINC01128 overexpression and ARID5B or BTF3 knockdown. **D** CCK-8 assay and **E** LDH assay of THP-1 cells after LINC01128 overexpression and ARID5B or BTF3 knockdown. **F** Propidium iodide (PI) staining in THP-1 cells after LINC01128 overexpression and ARID5B or BTF3 knockdown; original magnification, ×20. **G** Flow cytometry detected the percentage of double-positive THP-1 cells. IC, β2GPI/anti-β2GPI immune complex. Data information: Error bars represent means ± SD with at least three independent experiments. ns, not significant; *P<0.05; **P<0.01; ***P<0.001.


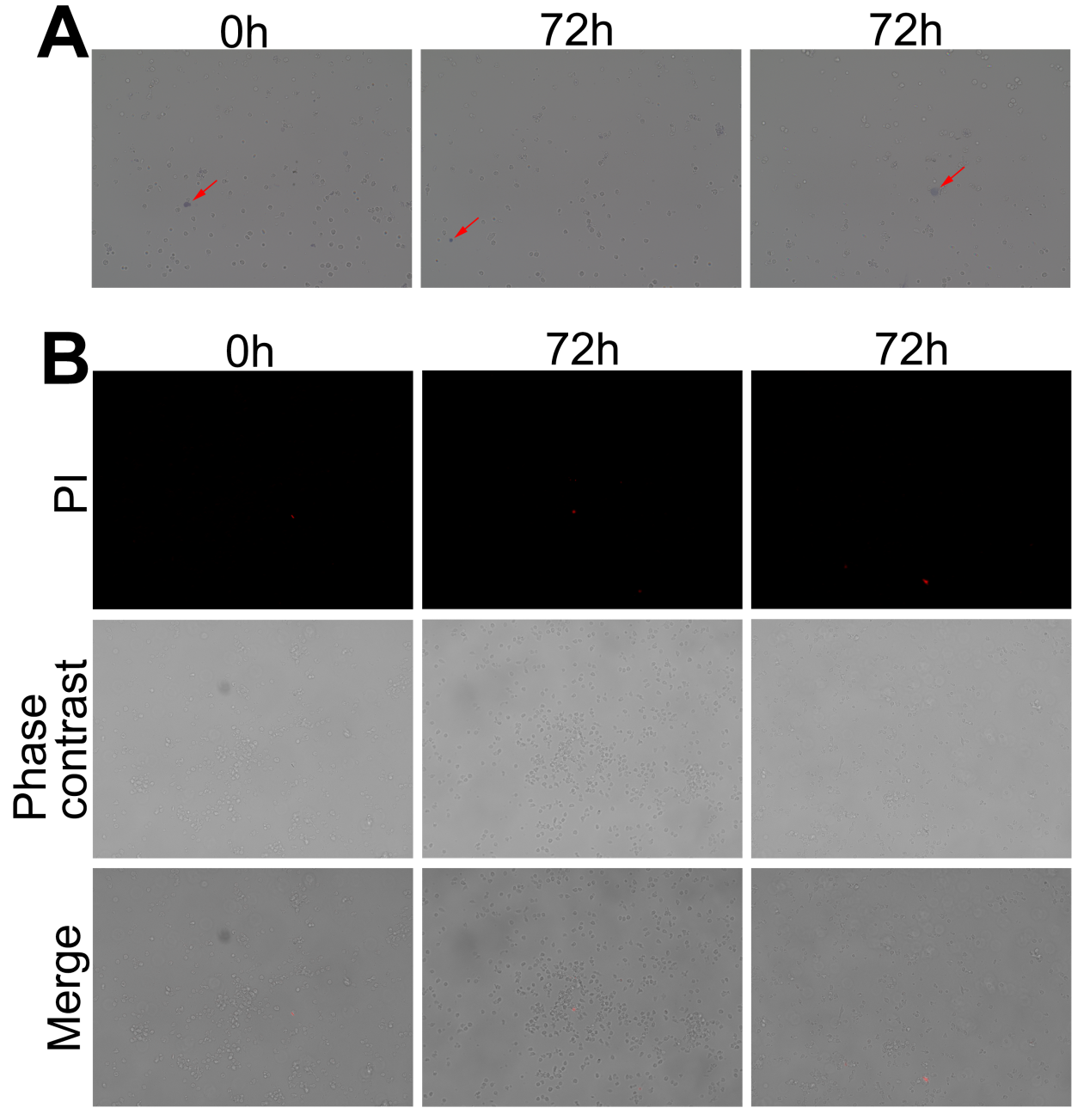


**Figure S4.** The validation of monocytes viability using trypan blue staining and PI staining. **A** The validation of monocytes viability using trypan blue staining at the beginning (0h) and end (72h) of the experiment. Red arrows represented trypan blue-positive cells. **B** The validation of cell viability using PI staining at the beginning (0h) and end (72h) of the experiment.
