# Supplementary material for: ARID5B‐mediated LINC01128 epigenetically activated pyroptosis and apoptosis by promoting the formation of the BTF3/STAT3 complex in β2GPI/anti‐β2GPI‐treated monocytes
Source: Clin Transl Med. 2024 Jan 15;14(1):e1539. doi: 10.1002/ctm2.1539 (PMC10788880; doi:10.1002/ctm2.1539)

**hTFtarget predicting the binding of ARID5B with lncRNAs**

1. **Predicting the binding of ARID5B with LINC01128-promoter**

LINC01128-promoter up2k

CCCTAGTGAAGTCCACACACATCCTTACATGGCAGCTGTGGGTATATCAACTGGTCTGACCCCTTTTAGTCACAGAGCCTGAAGTCTCTGCTAGTACCTGCTGAGCACAGGGTCATGGGTGAGAATGGGCAAGTCTTTTTCTTTCTCTGGTTCCTGAACTTCCCAGGCTCTCTCACTTCTGGATCCTGAATACCCAAAAACCAAGCTTCCTTCCCAGAACCAACACCTCCTCCTCATTAGAAAGATACCTTTGTTCTGTGCTTACTTTATAAAGTCTTGCTCTTTCCCTATCCACTGCCTTGTGTCAGTATGTGTGTGTCTTGGGGCCGGTGGAAAGGTGAACAGAAGCCAGTAGAGAGTAACCAGCACCAGCTTCACAGGAATGGCATGACCTTGATACGATAGCGGCAGTTTTCCCTTAACATCCCTGCCCCCTAAAGACTTCAGATCATATGTCACACTCTTAAGTAACAGCGATTTGTCTTTCACGGTTTTAATCATATTGATTAAAAGCATTTGTCTTCTCCAGAACATCACATTAAGTCATCAAAAAATATAAACCTTAAAAAGATGTAATTATTGGAACTGAAAAACATAAAAGGTGAGCTTTGGAAATATCTTTGAGCAATTTATTCGTTATGAACACATGTAATCTGTTGGAACAAAGTTCTAGGACAATCCAGGTCATCCTTCAATATTTGCAGAAAACATCACAAAGAAAACTTGATACTTATACTTAGTTGGAAACTTTATGCCACAGCCATTAAATACAGAGATCATACAAAGGAATAGAGGAAGCTGTTGAATACTAAACTAACTCAAATCATCAATATCCTTACGGAGTGCACACTGAATTAATGTAAAAAGCATTTAGTAAACAAAAAGTATTTTCAATATACAATTAAGACTGAAAAGTATTGAGGCCTCATAAGCTGAACCTGACACAATAAATTTAAAAGGGAAACTAATTTGGAAATCAGAAAACCACTAAGGAATTTGGGAATTAGGCTTCTGCTGCCCTCTCTGCTACTGACGGTCAAGGCCTCCTCATTGTATTCTGTCCTCCATATCTCTGCTGATTCCCATTTTGTCTATTTCCATTTACCCCACTACTGCTTGCTCAGGTCACTCTCCTTCACTGTCGGTGTTTGTTCAAATTCCTCAGACCCATCCACTTCCCATCTAAACTTCCCTCCCCTTTTCTCGCTCGTTGGCTCTACCTCCCTCCTCTCTGTTTTCTCCTCACTCTCCTGCCCCACCTCGACATCCACAGCGAGGCAATGAAGAAGCCCCTGCCAAGGAGGAGCCCGCTTCTCAGTGGGACACCGGGAAGGTAGACACCCAACAGTCACCGCTAGTGGGAGGCGATTGTGCAGAAGCACGAGGGTTGTTACAGGATCGGGCAGGTCCCCTACCCCAGTCTCGGACTCAGGGTCCTGTCTGAGGCGGCCACCCCGAAGCGTGGGGTTTGCGGAGACGTAGGGCCTGGCGGAGGGAAGGACGGGGAAGCATCTCAGGGAGGACTGGCGTCTGCCGAATCCCAGGGCTGCCCTGAGGGGCCAAGAGGGGCGAGGGTGGGGACGATGGAAGACAAGCCACATGCCGAATGGGGACCTGAGGCCGCGCGCGATAGGATGGGCGGACGGTGAAGAGAACTAGGGTGGAAGGGCCGGACAGGGGCGACCTCAGTGACGGAACCGGACACAGACGCAGATCTGGCAGCTGAGCGACAGGCTTCGGAGCATTTCCGGGCGTCGCGGGACTCCCCGCCGACAGGAGGGCGGTTGCCGAGCCTGTGACATCCGCGGAGACCAGCAGACCCCGGGTGTGGAGGACGCCGCAGGGAGGGGACTGCGTGGCTGGGTTTGGCCACAAAAAGCGGAGGGCACTCACCCGAGCGGACCTTGGCTCCGGATAATCCGTTTCCGGGTCAACAAAAAACGTCGCGCGAGGGGCGGGGCGCGTACGTGCAGGGAGGGGAGGCAGAGAAAAAGGCGGGG


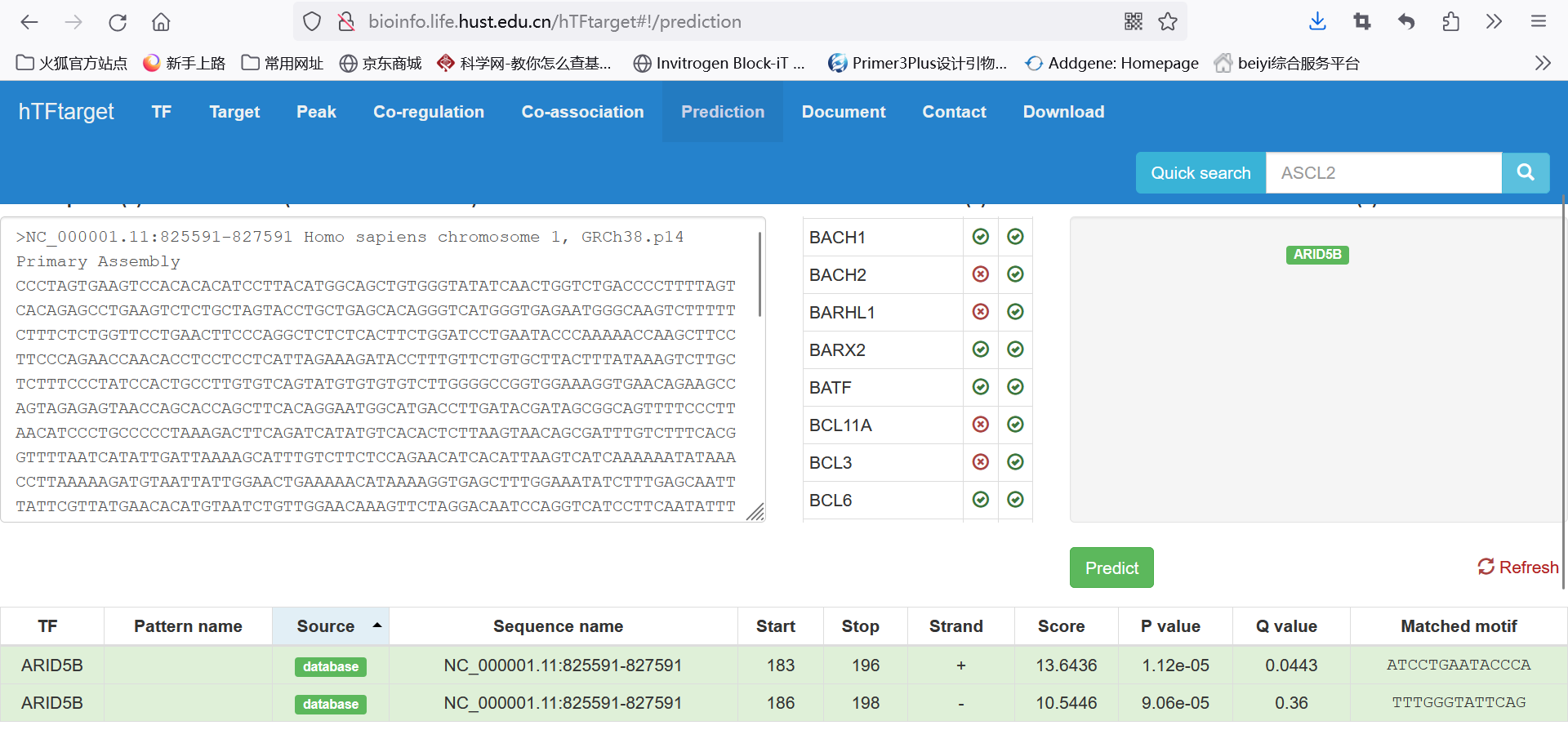


1. **Predicting the binding of ARID5B with LINC01125-promoter**

LINC01125-promoter up2k

>NC_000002.12:97661941-97663941 Homo sapiens chromosome 2, GRCh38.p14 Primary Assembly

CGAACCCTGCAAGGAAAAACAAAGTTGAGCTGCCCACATGCACCACCAGATGAAGCCAGTCCCCCGCAATGGAGAGAGCTGGCTGCCCCGTCAAGGCAGGCCAAGGAGGAAGCCAGGGACCCTGCCAGGATCATTTACAAGTACACCTATGGTTCAAGATTGGGGGCAATTTTGTATCCCCCTGCTCTGGCAACATTTGGCAATGTCTGGCAGCTGGGGAGAGGGGACATGCAGTGGGGGATGCTATTGGCATCTAGTGGGCAGAGGCCAAGGATGCTGCTCAACATCCTACGATGCACAGGCGAGCCTGCACTACCAACAAGGAGCTAGCCCAGAATGTCAATAATATAGAGATAGAGAAACCACGAAGGGCACCTTCCTCTGCACAACTAATGGTTGGCTCCAGCAGGATTCCATAGCACATGCTACTCCCAAAAGCTAGAAGAAACCCCTCTGTGTGGGTCTTTTTTTTTTTTTTTCAACAGCTTCCTTCTCCCCCAAGAACCCAGAAGGCATGGAACATGGACGACCTACAGGGCCTGCTGGAGAAGACCAATGGGTGCATGGGATGACCGGCAGCTTCCCTCAAGTGGCTTCCCAGAGACTACTAGGAGAACTTGGTCCTATCGCTGCCCCCACCTGGAAGCTGGACTTAAGGATCCCCCAAAGAACGGGGCAATTAGAAACCTCCCACCCAGCGAAGGGATAAGCTTCTCAACTCAGTCCCACCACTCTTCATCGCAACCCTCTGAGTCTGCAGCAGAAACAAACATCTCCAAGTTACAGAGGAGGGGATGGAATCCCCAAGGGGCCGAGCGGTAGCCCTTTTAACTTATAAGCCTGTTGATTAGCCTATACGAGTTATTTGCACGTCAAGAAAGGAAGTAGCCTGCTCCTTCCTGCAGCGTCCTGCTGGTGTGACAGCACGTCCCCAAGCTCAGTGCTAACCTCCTTATTAAACATCCCCTGCTGTGACTCAGGGAACCCACATGGGTACTCTAAAACAGTCATTCAGGGACCCCACGGGGTCATGTGGGAGGGAGACAGATCCCAGAAAGAGCACAAGTGAGTCATTACCAAAAACTCCAAGGCCCGCACACCGGACGCACATACCCAGCTAGGGGCAGACTCAAAGATCCCAGCCCTTATCTTCTCCCCATATCAGAGCTCGGAAGCCAGAAATCTTCCTAAGGCAGGTGAAAGCAAGCCGAGCCCCACTGCTGAAGGACAAAGCCACAGGAAGCCTGATGACATCTTTCCTCTGAGGCTTCCAAACGATCACCCCAAATTGCTTGCTGATACTGGGAAGAGTGGCCATGAACTCTCCATTGCTCTGCTGGCTGTGGAATGTTTGCTCAGCACAGGAAGCATTTAAGGAGAAAGTCAAAGTAGCCAAAAGGCAAACCAGATGGTGGTGGACATGTGGGTGACAGAGCATCCTGCATTTGTTGCCTCGGGGTGCAGCCCCAAAGATAAAGCCAGCAGTGTGCAAATGACAAATGCTACCCCACCTCCGCCAGGCAGCCAGAGCCAGGGCCGAAGGACGCGGAAAGGAACTGGTGTGGAAACCTGCCCAGGAACCGCACTCTCAACTGAGAAGAGTCCGGGGCGCGTCCCCGCCCGGCCGCCCGGCTGTCACTCAAGCTCTCCTGAACTTCCCCGCCCGACTCGGGGAGGGGTCGGGGAAGAGGCTCTGCGACGCGGGCGAGGGGGCTCGCCCCATGGCCGGCCTTACAAGGCCAAGAGGGCGCGCGCCCCCGGGGCGAAGCCAGCGGCCCGGCGGGGGCGACGGCCACGCAGTGAGCGGAGGACGCGCCGAGGCGGGCGGGGGTCTCTCCCTGCGCCGCCGCGTGCGCCCCCGGGGGCGGGGCGCCCGCCCTCCCCCTGGCTGCCGGGCCTCACGTTGTCGATGACCACAGGCTGGTTGGCGATGATGTCGTAGGACTCCATGGCCGGGCCGCGCCGGCCCTGCCCAGCAGGCGGGCTGCAGGAGGCACCGGA


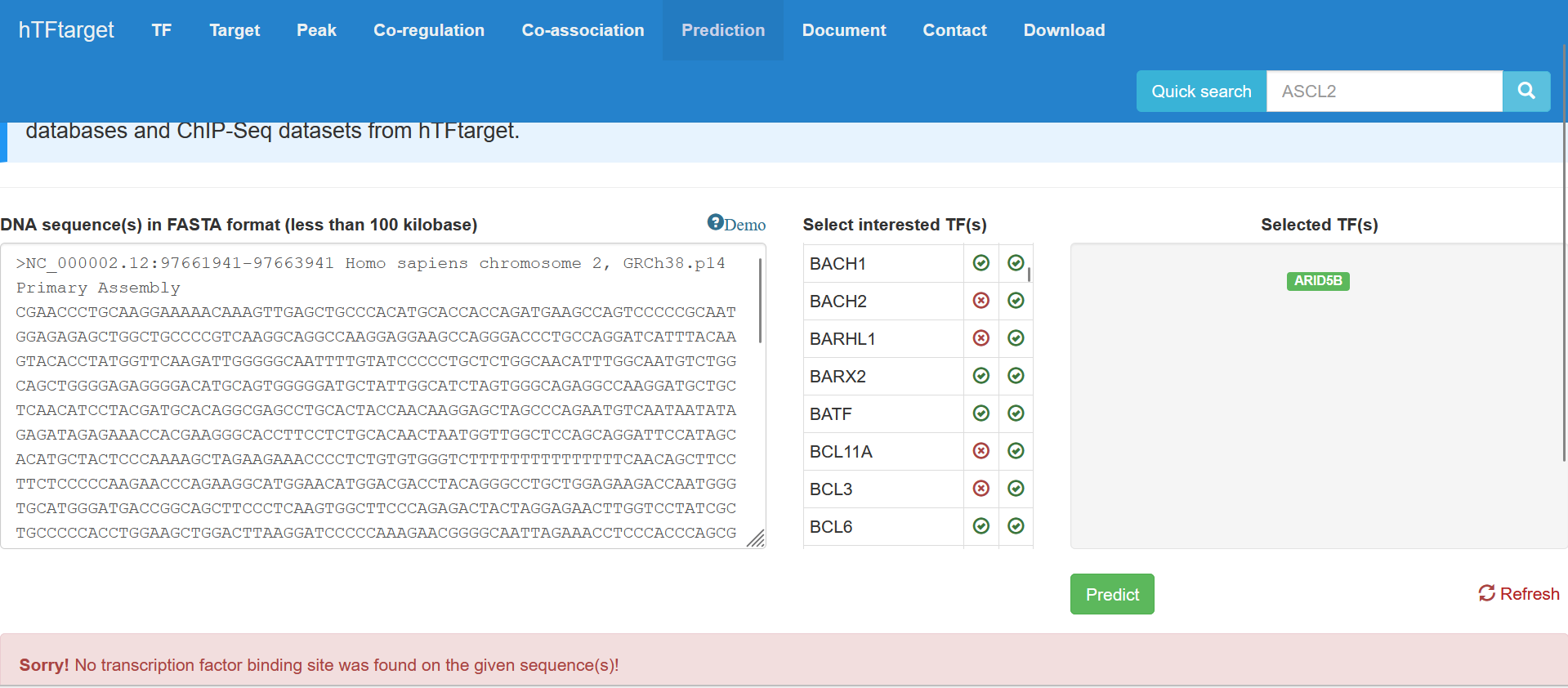


1. **Predicting the binding of ARID5B with LINC01624-promoter**

LINC01624-promoter up2k

>NC_000006.12:170270474-170272474 Homo sapiens chromosome 6, GRCh38.p14 Primary Assembly

TGGGGCCAGGGGACGTGTGGCATCATTTGCCCATGCCATTTTCACTTTGTTTTTTTTGTTTGTTTTTGTTTTTTTGAGACAGTCTCACCCTGTCACTCAGGTTGGAATGCAGTGGCACAATCTCAGCTCACTGCAACCTCTGCCTCCCAGGTTCAAGCAATTCTCCTGCCTCAGCCTCCCGAGTAGCTGGGATTACAGGTGCACACCACCATGCCTGGCTATTTTTATTTTATTTTATTATTATTATTATTTTTGAAATGGAGTCTCACTCTGTCACCCAGGCTGGAGTACAGTGGCATGATCTCAGCTCACTGCAACCTCTGCCTCCCGGGTTCAAGCAATTCTCCTGCCTCAGTCTCCCGAGTAGCTGGGACTACAGGCACACACCACCACACCCAGCTAATTTTTGTATTTTTAGTAGAAACAGTGTTTCACCATGTTGACCAGGCTGGTCTCAAACTCCTGACCTCAAGTGATCTACCCACCTCGGCCTCCCAAAGTGCTGGGATTACAGGCGTGAGCCACCACGCCCGGCCACCTTCTTCACTTTCATAGAATGTCTGGACTAAGTCTTCAAGGCACAAGGGCATCAAGCTCTAATGAGCACACGGGGGCACCACTGCAGATGGCCAGGGGGCAGGCGGAGGCCACCATGCACCCTCCCTCTTCATCGGCTCGCAGCCCCTCCTGGGGAGCTCTCGCTGCCATCACAAGCTGAGAGGCTTACAAGACACACATTCATCCCCTCACGGTTCTGCAGGTCAGGAGCCTGACAAGGGTCCCACTGGGGTAAAAAACCAAGGCTTGGCCAGCCTGGCTCCTTCCGGAGACTCCGAGAGAACCTCTTCATGTCTCTTCCCGTTTCCAGAGGCCACACATTCCTGGGCTCCTGGCCCCTTCCCTGTCTCTGAGCCACACTGCATCTCCAGCCTCGCTGACCCACCCCTGCCGCTGCCGAATTCCTCCCTCTGTCCCCTGTACAGACCCTGTTCTTCCACTGGGCTCCCCTGGGGAATCCAGGCTAATCCACATCTCAGGGCCTGGGGCTTAATCCACAACGTGCTGTTGGCTCTGGGGTCGGGAGGTTGGGGAGGCCGTGCCCGGGGGCCTTTCTTCTGTATTCCACACCATTGATCCACATCATGTTCAAATGATCTCCTAAATGGAATGGCTTCCTATCAACCCACGTCACTGGCTTCACCCCACTGTTACCAGGAGCTGGTTCTTGTTGGGCTCTGGAGGCCAAGAAGGGGCTCCTTCCCTCTGCCCATCAGCAGGTTGCTTGGCACGGTAGGGTGGGTGCCCCCGTGAGACACAGCTTGGGAGCACGAAGCGGCCGCAGGCCCTGGGCCCGGGCTACGGGCTGGGGAGGAGGGAGGATGAGGCGTGCAGACACTGAAAGGGCGGCTGCAGAGACTTTTTTGTCCAGAAGTAACCCAGAGCGAGCAAGTCCCTGTGGGTGAGGACCACGCATGCCCGGTTGGCGCTGATGTGTGGGAAAACGACAAATGGTGCAAGCCACACGGGTCACGTGCGTCTCCTGTGCTAATCGTGGATTTTCATTTCCTCCGGAGACCCAAATGAGAAAGTAAAAGTGTATGCAGGAGTCAAACCCACTTTCTAGAATGTGGTTAATTTCTAGAGGCAGCTGGGTTTCCTGTGGAGAAGACAGGTTTGCCTACGCAGCGTAGAGAGGAGACGGCTGTGTGGGCTCCAGCCCCAGGGGAAGTAGGGCCCGGGGGTCACCGCGAGGAAGCTCCATCGGCCGCGGCGGCTCTGTGGAATCTGAAGCGCGTGGTCTCACGGCCACCTCCCCCTGCACCAGCCGTGTGGCCCAAGGTGATGTCTGTGTGCCTTGGTTTCCTGATGCGTAGAACGTAGCCAGCTGGAGGACAGGGGGGCTGTCGGGTTCTTTGTGTGGAGGTCTCCAGAAGGCTGTTCGTGCTGTAGGCAAATTCCACAAGACCTAGGAGCCAGGACCCCAGGTGCCTTGATGCTGGA


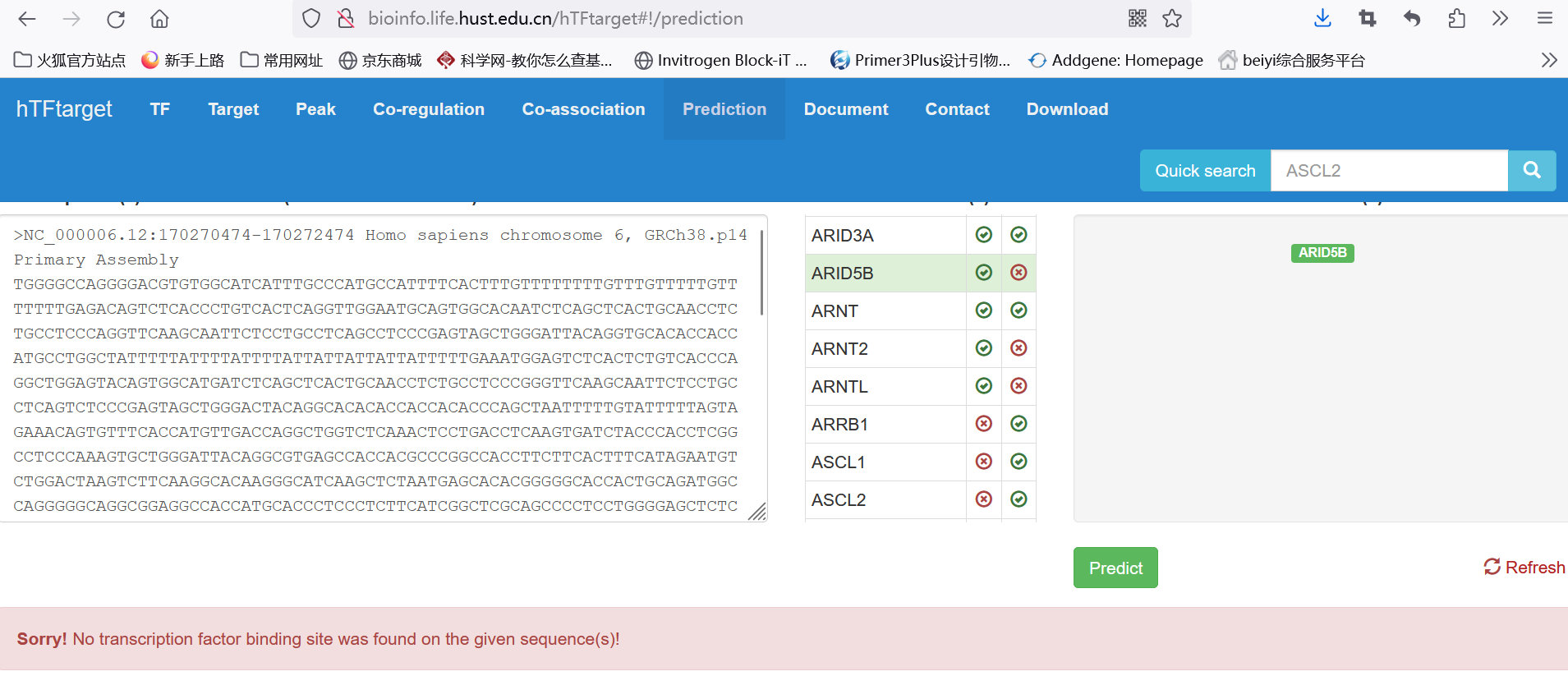


1. **Predicting the binding of ARID5B with LINC00685-promoter**

LINC00685-promoter up2k

>NC_000023.11:317145-319145 Homo sapiens chromosome X, GRCh38.p14 Primary Assembly

TCTCCCCGGAGAAGGCACCTTGAGCCGCCGTTTGTCCCCCGATATGACTTCGTCCCCCCAGGGCCTCTAA

GTAGGAATCTGACGGGAGAAGGACACCGGAGCCACAGCGGGACACGAGGGCGACCCTGCCTTACATGGAA

TTTGGGCTCCAGGCGGGCGCAGCAGGGAACCCGGGAGCTGCGGGTGCTCAGGGGATTACTGAGGAACCAC

CGGCAGGTTTGGGGAGTAGAGAGGCAAGAGGTCTGCGCGCTGTTCTGAGCAGTTCGGGGCTTCATCTGTT

CCAGGAATTAGCCATTTTGGAAAACACCCGTAAAGAAAAACCACTCATCGACTACAGGAGCTTCCAAGGG

AAAAATCAACCTTTATGAGACTCTGACACGTGCAGCGACTTATTCATTTGCATTTCCTCCTGGGGGGTGG

GGGGTGGGACTAGACACAGATTGGTCCGCTGGACGCCCTCCCTTCCCTGACCTCAAACACACCGTGCCTA

CCTCTAACCCAGAGCAAGAGAAACGTAGGTCCCATCGGCGAGAACCGCCCCGGCCCACCCAACCCCACCC

CACCCCACCCCACCCCACCCCACCCCACGGACCCCACGGGCCCCACCCCATCCCACGGACTCCACCCCAT

AAGCTCCGCCCCCGCACTTTTACCCCATAGGCCCCTCCCCCGAAGCCCCGCCCACGCACCTCCACCAATG

ATATCCTCCCCTCTTCCCCATAAGCCCCGCCCTTTCACCCATGGGCTCCTCCCCCGAACCCCACCCATGC

ACCTCCACCTATGAGATCCTCCCTTCAGAGCCCCGCCCCCGCATCACCATCCATAAGCTCTTCCCCTCAG

AGCCCCGCCCCTCAGCGCCACGCCCTGGCATCTCCAACCATCAGACCCTCTCCTTAGAGCCTGGCCCCCT

GAAGCCCCGCCCCTACGTCGCCACCTGAGCTCTTCCCCTCAAAGCCCCGCCCTGGGTCTCCACCTATGAG

ATTCTCAGGTCAGAGCCCCGTCCCTGAATCTCTACCTATGAGCCTCCCCAGAGCCCCGCCCTTGCATCGC

CAGCTATGATTCTCCCGAGTCCCGCCCCTGAATCTCCACCTATGAAATCCTTCCTGCAGAGCCCCGCCCT

TGCGCCTCCACCTATGAGATTCTCCCCACAGAACCCCGCCCCTGAATCTCCACCTATGAGATCGTCCCTT

CAGAGCCCCGCCCCTGAATCTCCACCTATGAGCCCCCTCCCCTCAGAGCCCCGCCCCCAGGTCTCCAGCT

CCTGTTTCTCCCCCGAAAGCCCTGCCGCGGTCACCTCGAGTCATCTGCGACTTCCCCGGGCCCCACTTGA

CGTCAGGGTGAACCAGACACACGCGCTGGGTCCCCGCCGGCAGCAGAGGCTCTCCCCGCAGCAGCTCCTC

CTCGGCGTTCTCGTCCGCATCTTCCGGCTCCTCCTCGTCGTCTCCCGTGCGGCTTCGGCCGCCGTCCGCC

CGCAGGCCCCGCCCTCCGCCCCACGGCCCCTCCAGATTCCCGGGGCTCCTGCGGCCGACAGCGGCTAGCG

CGCGCGCGGGGCAGGACGGCGCGGCTGCCCGCGGAGCCGAGCGGCCGCGGCCCACGCGGGAGAGCCGCAG

CCCCGGGCGTACGGCGGCCCGCAGGGCCCACATGGCGCGTCTGGAGGCTGAACGTTGGGGCGGGGGCTGC

CCACAGGCCGCTCCTCATCACGTGCTCGTGATTGGCGTGCGGCCGGGGCTGGCTCACCAGCGCCTCTCCC

CGGCACAGGCCGTGCTCCTCATTGGTCATCCGGCGTCACGTGCTAGTGATTGGCGTGCAGCTGGGGCGTG

CTCACCAGCGCCTCTCCGCAGCATAGGGCGTGATCCTCATTGGTCATCCCTTGTCACGTGCTGGTGATTG

GCGTGCGGCCGGGGCGGGCTCCCTAGCGCCGCGCCCCGGCAGGGCGTGCTCCTCATTGGTCGCCCCGCAT

CAGGTGCTGGTGATTGGCACGCGCTGCCGGCGGGGGAGGCG


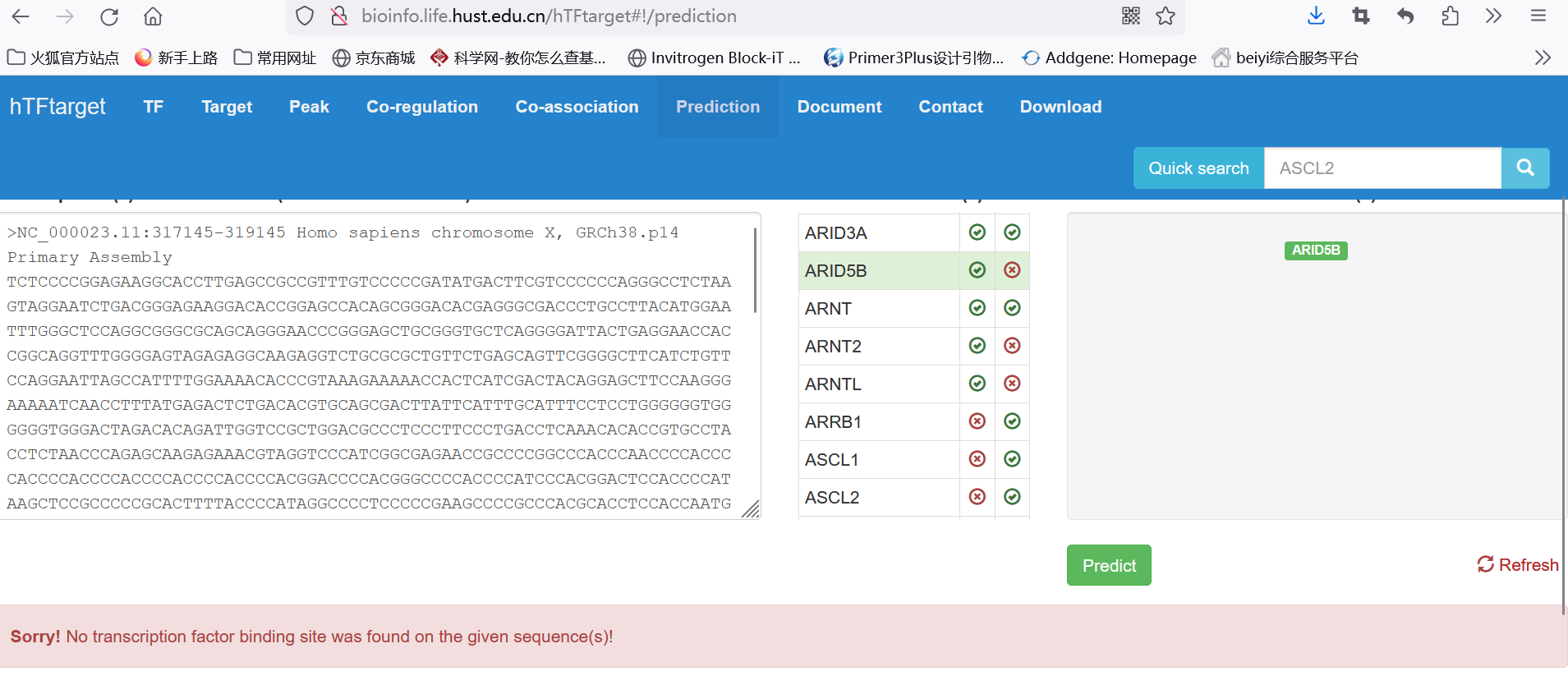


1. **Predicting the binding of ARID5B with LINC01300-promoter**

LINC01300-promoter up2k

>NC_000008.11:141338549-141340549 Homo sapiens chromosome 8, GRCh38.p14 Primary Assembly

GGGTGATGCTTAACCCAAGAGTAGGTGCTGTCAGGCCGAAACATCTGTTCTGGTCCTTGGCTGGCTTTCC

CAAGGCCCTGAGTGTCCTAGGGCCTTGTGACTTCCTCTGTGAACTGGAAAACAGCCCCCAACTCCAGTCC

CTGCCTGGCTAGGCTTGCCTGAGCAGAGCAGAGGTGAGAGGCAGGAAAGATCCAGCAGCCTCAGAGCTGT

GAACGGTGCCAGCCCCGTGATTGACATGTCAGCCGCACTAATAGTATCGCTTCATCTCTCCAGACTCTCC

CTTTCCCGGTTTGTCCACTGTTCTGTTTGGAAATAAGGTTGTGCCCTTAGTCATACCTTGATAAGAAGGG

AGACGCCCCTGCCTGCCCTCTCTCTTCTGTTCCACCTCCTCTGCCGAACGTTCAGGAGCTCCTCATTCAA

AAGACCGTCAGATGAAATGTTTGCTAGAGATAACACTGTGAAGCATTTAGAAACACCTTGCACCAGCCTA

CTTCTTAAATACTTGTTAAAAACGAGATCACACCCCAAATAGAGTTTCATAGAATATACCTTTTTTATTT

TTCATTATTTTTTGAGATGGAGTCTCACTCTGTTGCCCAAGCTGGAGTGCAGTGGCGCAATCTCAGCTCA

CTGCAACCTCTGCCTCCAGATTTCAAGTGATTCTCCTGCCTCAGCCTCCCAAGTAGCTGGAATTACACGC

ATGTGCCACCACACCTGGCTAATTTTTGTATTTTTAGTAAAGACATGTTTTCACCATGTTGGTCAGGCTG

GTCTTGAACTCCTGACCTCAAGTGATCTGCCCACCTTGGCCTCCCAAAGTGCTAGGATTACAAGCATGAG

CCACCGTGCCTGGCCTCTTTTTTAAATCCAGTTTTTATTCAGCAGAATGTTCCGAAAGATCCAGAGGGTA

ACATGTGGGCAAAGTCCCTGAGCCCTGGCTGGGGTGGCCTGGGTGGGGTTGCCTTCCTCCGACACCCAAC

TCATGTGGGCCCTGGGCGCCCAGCACCGGGTTCCTTCCCCCCAAACCCATCTCATCTGGACTCTGGGCAC

CCAGCGCCGGGTTCCTTCCCCCAGGCCTTGAGCCTGGCTTTGCAGCTCCTGCTGCCCCAGGGCAGGTGGT

GGTGGCCGCCCAGCCCTCACCCAGCCCCGAGTCCTGTGTGGAAGGCGATTCCTCCCACTTTACCGGGGAG

GACACAGAGGCTGAGCGTGGTCACGGCCGGAGCCCCAGCGCAGCATCTGAGCTGCAGGCTGGCCGCCCCC

CACCCCGCTGCACCCCCATATTCCACTCACAGACCTTCTTCCTGCTGAACACAGCTGGGCCCTGACGCCT

CTGTGCCTTGGCCACAAGGCACCTTCTGCTCACTCAGCTGTCCCTCCAGTCCCCGTAGGGCTGCAGACCC

AGGCCCACCCAGTCACAGGCCTGGCAGCGAGGGGGAACAAAAGGGTCAAGGGGCCGCCCAGGGCTGGGAA

GGGCCTTGCTCCACCTCGCTCTCCTCCATTCCCCGTGTGGGCCTCCGTCTCCCATTCTGTGAAATGACCC

GTTTGGGCCACAGGGTCCCCAAGTCCTCTTGGCTTGGGGCCGTCTGTTCCAAGAGGAGGGGCCATATTGG

TCCTTGAGGCCCCTGCGGCCCAGTAAGTCTGCAGTGAGGCCTCCCTGGACGTATATATCAACAAAAAGTC

AAACTCCGTAAAGTGTTTGAGACATTTATTCTGAGCCAAAGATGAATGGCCGGGGGCCCATGACAGCCCC

AGGAGACCCTCAGACCATGTGTTCAAGGTGGTCGGGCCACAGCTAGGTTTCATACATTTTAGGGGGACAC

AAGGCATCAATCAATACACATAAGCTGTACATTGGTTACGCCTAGAAAGGCGGAACCACCGGAAGCGGGG

ATGCTTCCAGGTCATAGGTGGATTCGAAGATTTTCTGATTGGCCGTTGGTTGAAAGAGTTATTGTCGGTA

GATAGGAATGTCTGGGTTAAGATAATGGGTTGTGCAGACCG


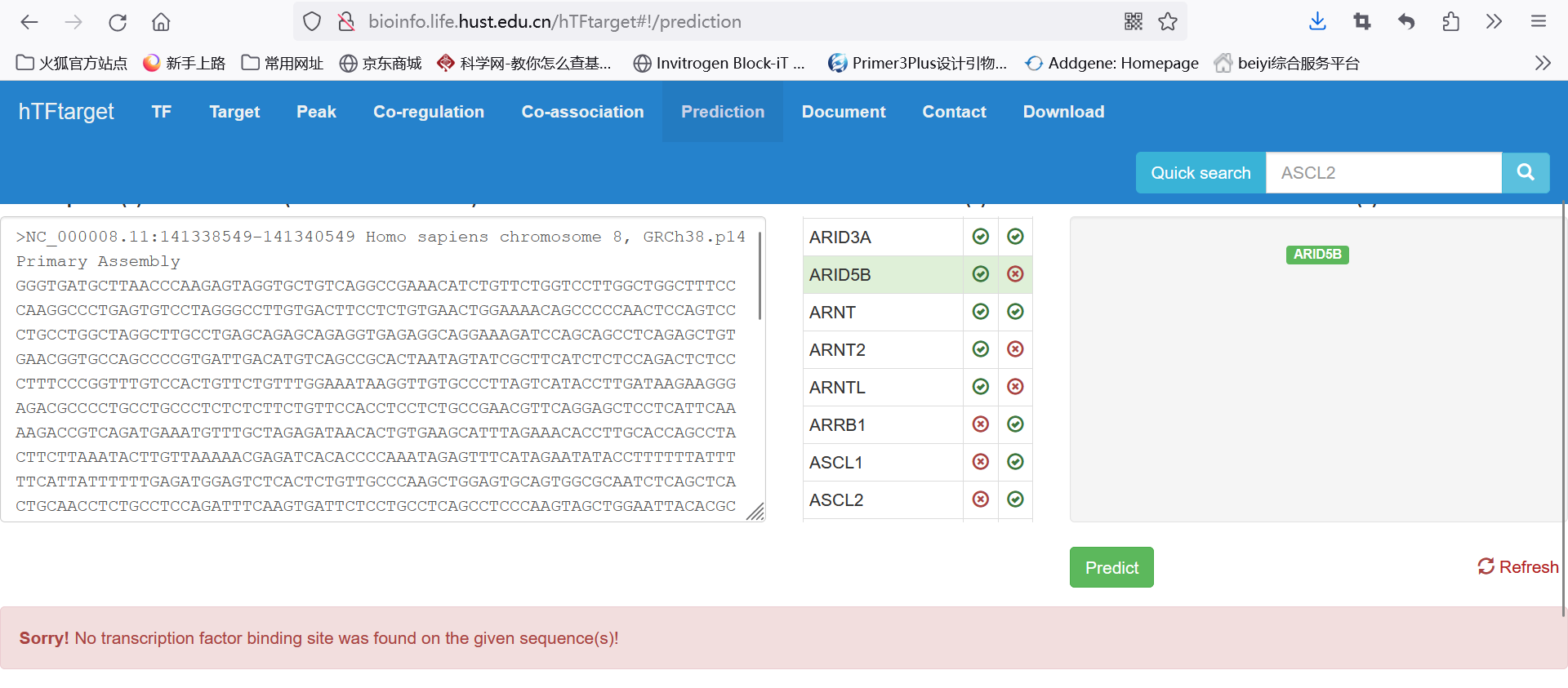


1. **Predicting the binding of ARID5B with LINC00167-promoter**

LINC00167-promoter up2k

>NC_000011.10:130000624-130002624 Homo sapiens chromosome 11, GRCh38.p14 Primary Assembly

TTTGAAAAAGCAAAACAATTTTGCCTTGCAAAACGAGGAACAGCATAGCAGAGTGGGAGCTTACGGGGTA

GAAGGCACAAGACATTTGTCTTGATCCAAAACAAACAAAAATGTAATCCTTTCACTAAACGTCCCTGTTC

ACTTTAAAATGGTTCATTCTGTGTCAACTTCACCTCAATTTATTGTAAAAGTAAACCTAAAGGCCGAGCG

CGGGGGCCCATGCCTGCAATCCCAGCACTTAGGGATGCTGCGGCGGTAGGATTGCTTGAGCTCAGCAGTT

CGTAACCATCCTGGGCAAAATGATGAAACCCCGTTTCTACAAAAAGTAGAAAAATTAGCCAGGCGTGGTG

GTGCACGCCTGTAGTCCCAGCTACTAGGGAGGCTGAAGCAGGAGGATCCCTTGAGCCCGGGAGGTCGAGT

CGGCAGTGAGCCAAGATCGCGCCACTGCACTCTAGCCTGGGCAACCAGAGACCCTGTCTCCTAAATAAAC

CTAGCAAAGTTCCTTTCTAAATACCCCATATTGAATAAACACTGTTTAAACGCTGCTTTGCCCCCAACCA

ATCCTAATTTTAAAAAAAAATCTTGTTTTCTCTATTCTAGAACAACAAATAAGTACTGCTCATCAAAGTG

GAAGCTATTGTGATGCTGGAATGGTAGGAAGATACAAAAAAAATACAATGTAATAATACACCTTCTTGCC

TTACAGCGATGGAGGAGTGATAGAAACAAAGAGAACAGGTCAGAAAAAGAGGCCACAAAACAGTCATGAG

TGATTCTGCTGTTTTTAATAAAGGTTAAGTACTGCTAAGTTTAGAGAATTTGGGGTGACGAGACAGGCAA

GGTTTCAACAGGCAAAGCAGCAGAATTAGGGCACAGGGCAGAGCTGGGTGGGTCTCATCACACATCTAAT

CCTCGATGACAGCACAAAGCCACCTCTCCTTGTGCTTAAGTGTACACGCACATTGATTGAAAATGCACTA

AAATCCAACTCATCCACCAAGCAAGGCTAAAGGCGCGCACACAAATTCAACAGCGCTGCGTTCGCGTGGC

TGGAGGGCAGACGGGGAATATTCCATTTTACTTAGTAGAGTATCAACACCTAGCTGCAGGGTTAAACCTG

CTTAACAAAAGCTGGCGAGGGGCTGGAGAGGTGCACACCATCAGCCCTCTCCCGAGCGCCGACCGGCCTC

GCCCTGCCTCTGCGCTGCACCCTGGGTGCAGGCGGACTGGGGCAGGGCCGCGACCCCGGCCTCGGCCTCC

GCCTCCGCCCGGCCAGCCCCGACGCGGTGCCACGGGACAGACCCGCAGGGGCCACCCCACACCCCTGCGG

GCCGGCCCCCCGCCCCGGTCCCGGACGCTAGCTTGGGAAGGCAGGCCGGGGGCCCAGCCATCGATGGCAA

GTGGGCGGCGCGGCCTCCGCAGACAAAGCTGGGCGCCCCATCGAACAGGACCCTCCGCCCCACGCGCCCG

CAACCGGCCAGCCCCGGAGCCCTGCCCGCACTGCCACCCCCAGCCCGGCGCCCGGCCCCCAGCCCCCGGT

GCTCGGCTCCCGGCCGGCGGAGACCTGCCCGCCGCGCCCGGAGCGCCCGCGCAACCGCCGCCGCCGGAGC

GCGCAGGCCGCCCAACCGCCGTCCCGGCCCAACCAACGCGGGCAGGGGAGGAGAGGTGCGCGCGGCCGGC

GGAGCGTGCGAGCCACTCGGGGGTCGCAGGGGGAGGCTGCGCGCGGCGAAGGCAGGGGCGACGAGACGCC

GAGGCCGGCGCTGAACGCGGCGCCAGGAGGGCCTGCTGGAGGGGGCCTTGCCCTCTTCCCCTTGGAAGGT

GCAGATGGTTTGACCCCCCACCCCGAGTGAGGTGCCTCGTCCCAGCCCCGGCTGGACTGTACCATCGGGC

GGTGCCGCCGGGATTTCTCCCCCCCACCACCACCATCAATTCCCCCCCCACCCGGGTCCGCGGTCGGTTG

GCCCCGGCCGGGCTCTGAGACGCGGAAAGAGCTGGGCGCCA


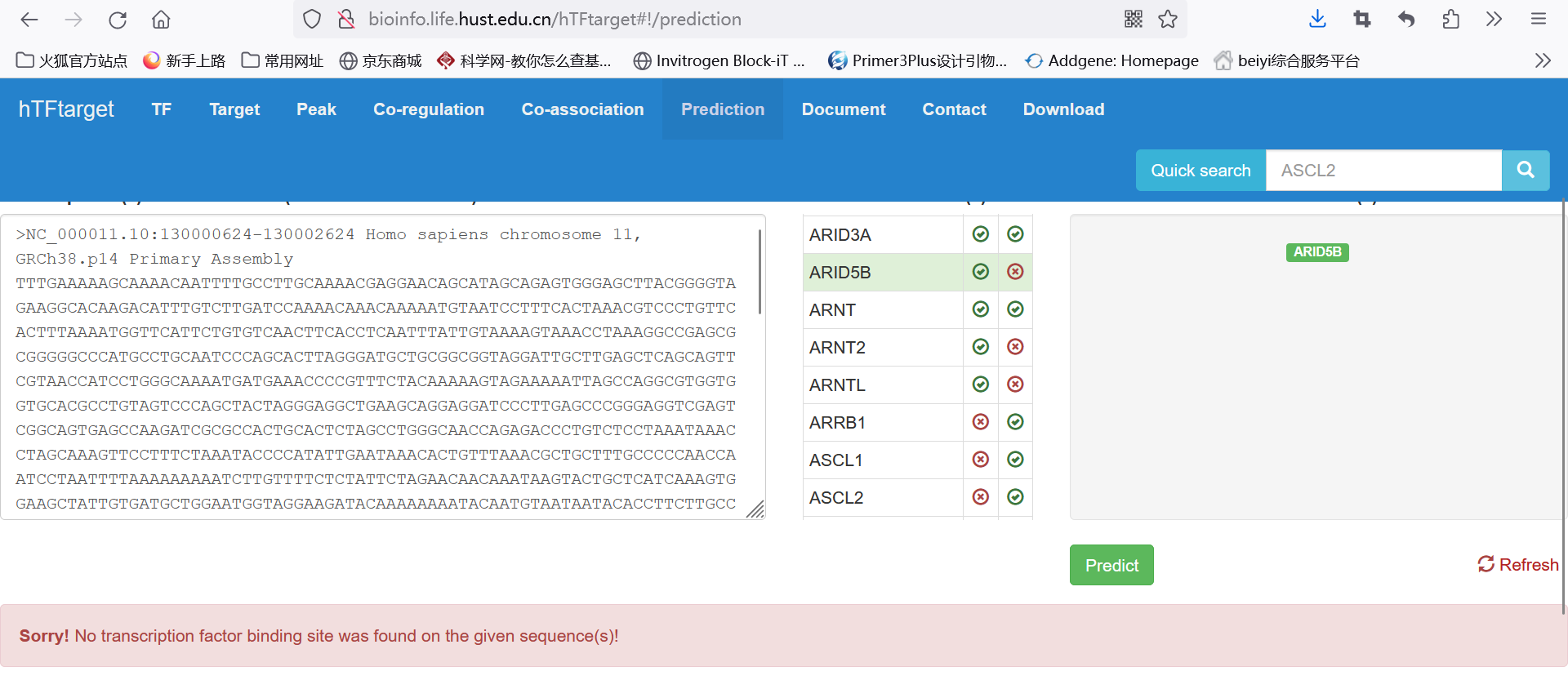


1. **Predicting the binding of ARID5B with LINC01585-promoter**

LINC01585-promoter up2k

>NC_000015.10:90658234-90660234 Homo sapiens chromosome 15, GRCh38.p14 Primary Assembly

ATGCTATGTGTCTGACTTTCGGGAATTTATTAGAGGGAGTGGAAATTCTACCACCTCCTATTGCTAAATC

TCATTAAAATGCCCTTGAAATCAACCCAAACCAACTCTCAGGCACAGCCAGAACAACGGAGGCAGAGCAG

AGCTCAGGATCCTGAATCCACACCCCCCGGCCACTCCTGGTGGGAAGAGAAGAAGAGGAGAGCTCGCTTC

CCACCATCCCCATCCCACCTGGCTGGAGAGGCTGATGTGGGGCGAGGTTTGGCCATCAGTCCACAGAGGG

GACTTCATGTACACAATTCATTAACATTTATTGAGCACCTATGATGTGCCAGGCACTGTTCTAGGTCCTG

AGGATATGGTGATGAACAAGATCCCTGCTCTCATAGGTTACCCAGTGGGTGCCCTAAGCAGGCAATAAAT

AAATATGTGAGCAAGATGACAAGAGAATAGCAGAAGGCACTAAAGTTTTGGAGTAAAGCCATGAACTCTT

TTTTTTTTTTTTTTTTTTTGAGACAGAGTCTTGCTCTGTCACCCAGGCTGGAGTGCAGTGGTGCGATCTC

GGCTCACCACAACGTCTGCCTCCCGGATTCAAGCGATTCTCCTGCCTCAGCCTCCCGAGTGGCTGGGATT

ATAGGCACGTGCCACCACACCCAGCTAATTTTTCTATTTTTAGTAGAGATGGGGTTTCACCGTGTTGGTC

AGGCTGGTCTCGAACTCCTGACCTCGTGATCCACCAGCCTCAGCCTGCCAAAGTGTTGGAATTACAGGCG

TGAGCCACCGCGCCCGGCCCGAAAGCCATGAACTCTTTTCCCAATTATTTTCTTATTGAGAAGCATCTCT

CAGCTTGCTGTTGGGTTCTGCTGTTAGTAAGCGTAAGGATGGAGGAAGGCTGGCTGATGACATTAAGCAG

CTAAAGGCGTGGACTGTCGAGGCCAAATAGCATCAGCTCTCTTTGGCTTCCCCAGCCCATGGAACAGTCT

TCCTATTTTGCGAATTTCCTACCTTATGAACTCACACCTGCTTATGATGGAAGCCAGAACCTTTCCCCAT

CTCTCTTGAAGATTGGGCACAGCATACAACTTAAGACACCACCCGTTGGCTGCTCCTGCATGAGGCTAAG

TGACACTGAGAAGCAGATGACTGGAGTCCTTTCTGTCAGGATGACAGCAGATCCCAGAGGCAGCAGCAGA

AAGAACCAGTGCAGGTAAATGGCCTTTGAGCCTGGTTCTCTGATTTCGCTAAGATTTTATGAGGCACCTC

CTATCCTTTAATAGGTTCCTCTGTAGCTTAAAAGCAGCTAAAGAGCAGTCTGTTGCTTGTAATTAAGGAC

TCCTACCAGTACACGAAGGGAGAAAAGCAATAATCAAATAAATTACTTTCAGATAGAGATCAGTGCTAGG

AGGAGGTCAGCACAAGGTGATGTAAGACAGAGTGACAGGTGGGGTGAGGGCTACACTTGATAAGGGGTCA

GTGAAGCCTCTCTGAGGAGGTGACGTTAGGCTGAGACCCAAACAATGAGAAGGAACTAGCCATAGAAAGA

TCTGGGCGAGGAGACTTCTAGACACGGGGAGCAGAAAATGTAAAGGATGAGCTTCAGATGTTGATGGATG

TAGGGAGGGTTGATGTGTTTGGAGTTTAGTGCAGGTGAATAAAGAGGAACATAATGAGATTGGAAAGTAG

GTAGGAGTCAGTTCATGCCAGGCCTTGCAGATCATGGTAAGACATTTGGATTTTATTCTGAGGTTAGTGG

GAAGCCATTAGAGAGTTTTAAACAGGACATAATATAGTACCTGGGGTAGCCAGCCTCCAAAACATCCTCC

AGTGATCCTTGCATCCTTGTATTTACACTCTTGTGTAGCCCCTCTCACATTGACTAGGGCTGACCCAATA

GGATGCTGCAATGTGACGGTATATAACTTTCAAGGATAGGTCATAAAAGACATTGTGACTCTGCCTTGGT

CTCTCAAATGTCTCCCTCTGGGGAAGCCAGCCACCATGTTG


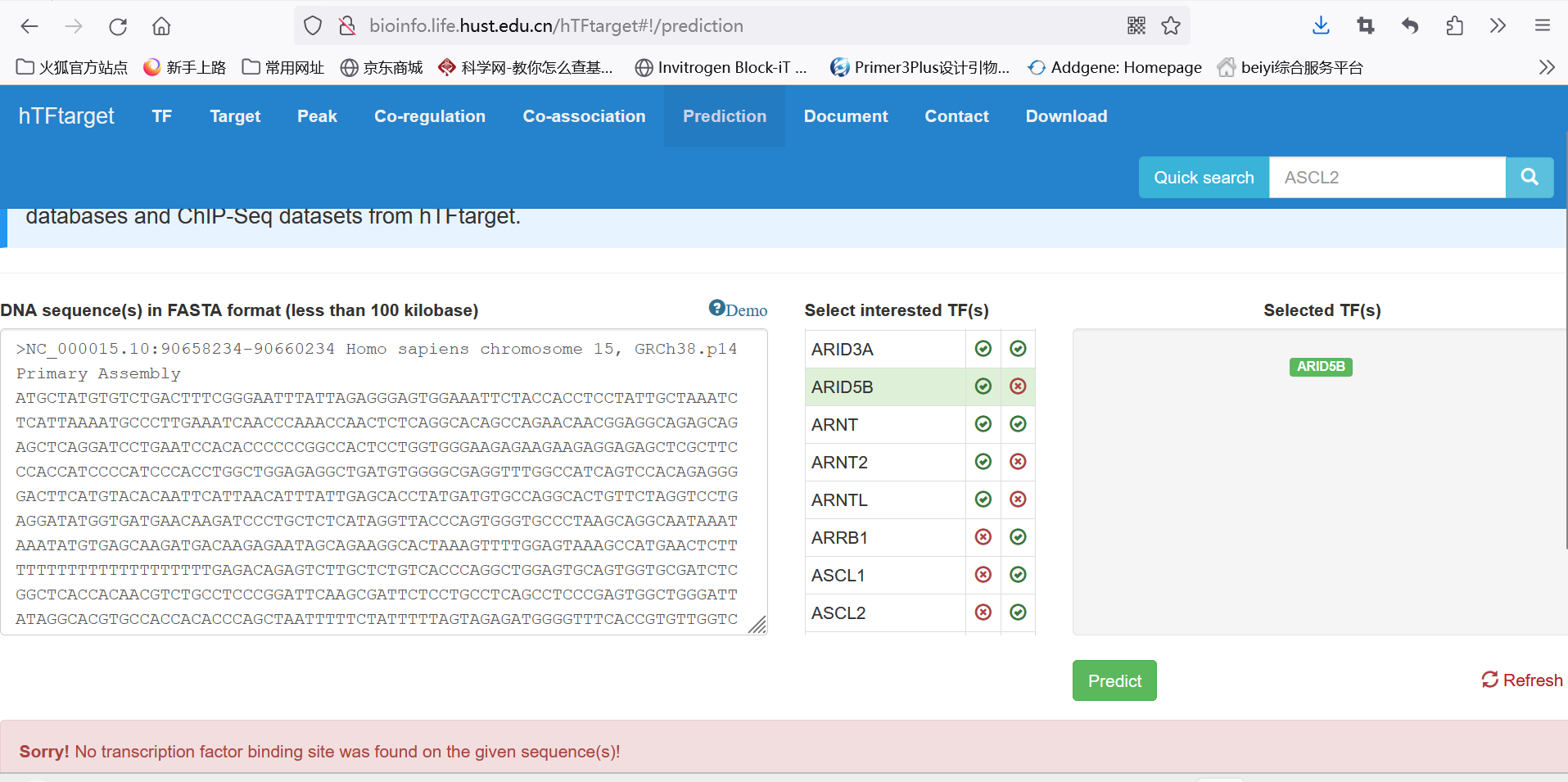


1. **Predicting the binding of ARID5B with LINC00176-promoter**

LINC00176-promoter up2k

>NC_051338.1:168769350-168771350 Rattus norvegicus strain BN/NHsdMcwi chromosome 3, mRatBN7.2, whole genome shotgun sequence

ATTGTCCTCCCTGCTGTACACTACAGTAACTACCAGGCGGGGAGGAAGGTGCCTAATTGGGTTTCTTTTTTGTTGTTGCTTTGTTTTTTCGAGACAGGGTTTCTCTATGTAGCCATCTTGTGACTCTGTAGACCAGCAGGCCTTGAACTCAGAAATCTACCTGCCTTTGCTTCCTGGGTGCTGGGATTAAAGGCCTGTGCCACCATGCCCCACTTCTAAGTTCGTTTTTAACAGTCCAGTTTCTTGGCTTCCTGCAAGACTCTTCTTCAGGTTGCTGTTCAAGTGTCTATGAGTATCAGGTACAACAGTCGTGTGCTATGGTCTGAGTGCCATGGAACTGCAACTCCAGTGGGCTGAGGTCCCCAGAACTGACCTGTGGAAGCCGATTTTTGTGTTTGCTTAGTTTGCTACAAGGCGACAAAGCAGGTTAATAGGGGTTGGTATCTTATTAAGGAGCAGTCTGAAATTATAGTTTTTTTTAAATCTGCCTTAATTAGTTAATTAAGGTTAGTTTCAAATTGTATTCAGCCCAGATAGTTTTAAAAAGTTAAGATCTAGCTGGATGGTGATGGTGGTGCATGCCTTTAATCCCAGCACTCAAGAGGCAGAGGCAGGCATATTTCTGTGACTTCCAGACCAGCCTGGTCTACAGAGTGAATCCCAGGACAGCCAGGACAGTTGCACAGAGAAACCCTATCTTGATGATGATGATGATGATGATGATGATGATGATGATGATGATGATGATGATGATGATGAAGAAGAATAATTATAATAAATAAATAAAGATGTTAAGATTCCACTGTGTTCTTAAAGAGCAGCTTGGACCACTGAAGCATCTGGAATGACCTCCAGGTAGCCCTGAACCATGACCCAGACCTTCTGTAACATATGCCCAGGCTTGCTGGTCTGACCAGGAGCAGGGCCACCTACATAGAAGAATGACTCCCAAACAGGGCAGACACTATTCTATGCCCCTTGCTCTAAGTACTTCTCCCATAACAGGCTGTTCTGGAGTGAAAGAAAGATCACCAAGGCTCGGGAGTGGTTCCATCGCACCGTGAAGATTGATTCAGATCTGGGGGATGCCTGGGCCTTCTTCTACAAATTTGAACTACAGCATGGCACTGAGGTGAGGCTACAATAGCACACCATGTGCATTAATAATCTTCCTGTTTCCTTAGCCACTCCTGAAACAGTAGAAAGCTCCATCTAATCCCTAAGAAAAGAAGGCAGGGAGTGGCCAAGACTAGCAGCCCCTCCCCTGTTGAGCTGTGTTGGAACCATGCTCATAAGTCAGGGGCCCCGGGTGTCCTGGGTTCCTTGTTGGGAGTAGTAGCCAGACTACATTGAATCCAGGAAATACCTGATTCAGAGATAGTGCACCTGGCCATTTGTGGGAATCAAAAGATAGGGAGTCTAACACAAGTTAACACAGAGACACACTGTCTTGGAGGGCATGTTCTACAAGAGAAAGAGATGGCTTGAAATCTGCTGGGGCCAGATCTGCAATGGTACAGGCAAGACCTGTACCGCACAAGATGTAAACCTCACAAGGAATCCGCCTAAAGTACCCTTTGGTCTTCCCAGGAGCAACAGGAAGAGGTGAGGAAACGCTGTGAGAATGCAGAGCCCCGACATGGAGAGCTGTGGTGTGCTGTGTCCAAGGATATCACCAACTGGCAGAGAAAGATTGGGGAAATCCTAGTGCTGGTGGCCGCCCGTATCAAAAACACCTTCTGATAGCAGGGGCGGAGGACAAGGCACTTTGGGGCAGCACATGGACAATGAGCACAAAACCTACACTGTATCTTTTATTCATTAAAGATTTTTATAGAGTTGTGTTGGGGCTGGGGCCTCATTTGTTTTTCCATTTTTGTCTTTGGTGTCTGTTCCCATGAATGATGGTCGTGTCAAGTTGGTTTGGATGTGGCCGCCTGCAAGTGCCTATGTGCTCCCATAGAAGCATAATTTGTGAAGATGTTAAGAGTACAGGCTATA


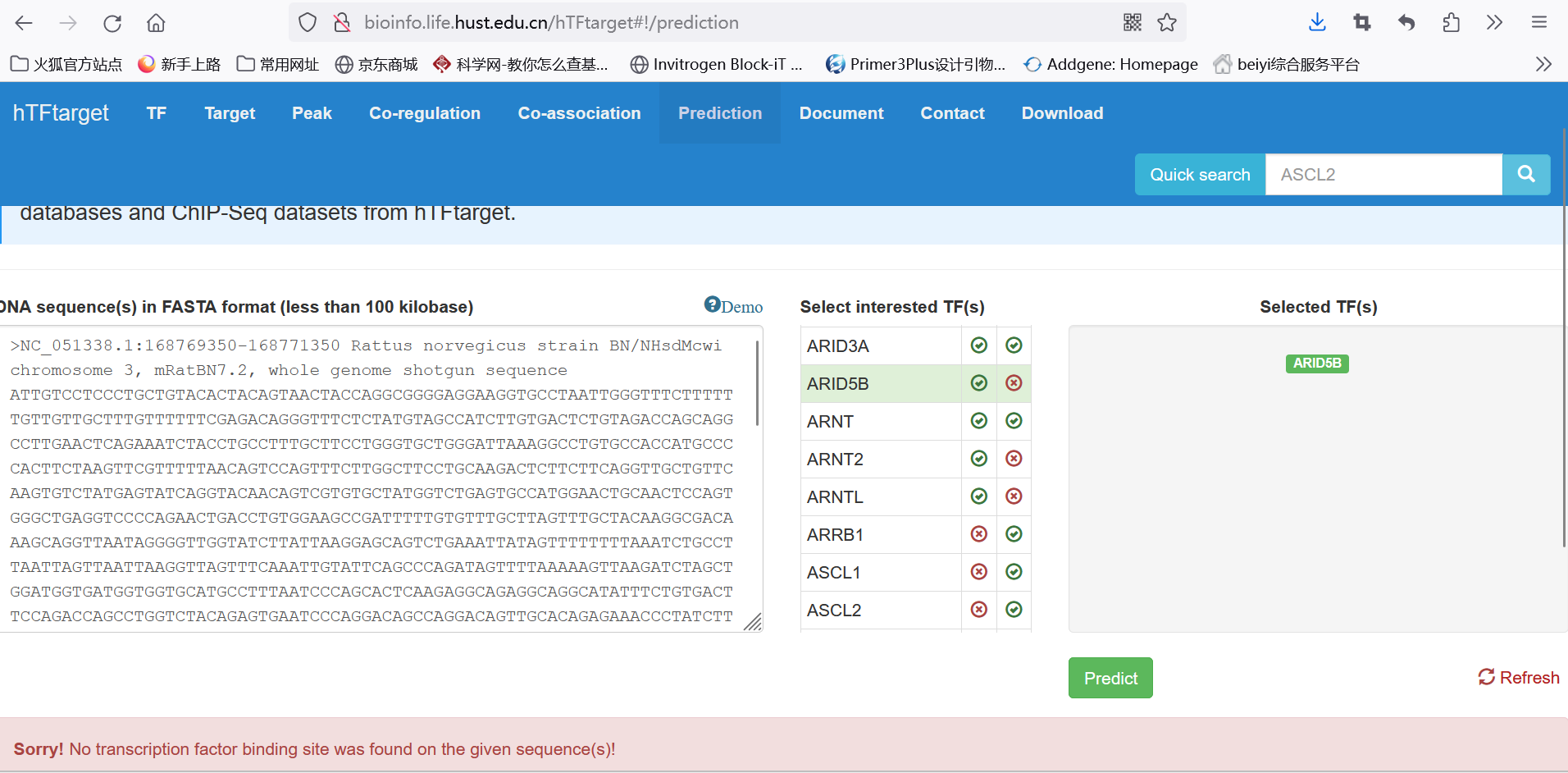

Supplement: Supplementary file 3 — Supporting information [file CTM2-14-e1539-s003.docx]
