# Supplementary material for: ARID5B‐mediated LINC01128 epigenetically activated pyroptosis and apoptosis by promoting the formation of the BTF3/STAT3 complex in β2GPI/anti‐β2GPI‐treated monocytes
Source: Clin Transl Med. 2024 Jan 15;14(1):e1539. doi: 10.1002/ctm2.1539 (PMC10788880; doi:10.1002/ctm2.1539)
Supplement: Supplementary file 4 — Supporting information [file CTM2-14-e1539-s002.docx]

**Table S1 The correlation between clinical features and aPLs in PAPS**

| **Clinical features** | **Triple positivity; N=12** | **Non-triple positivity; N=52** | ***P*** | Normal reference values |
| --- | --- | --- | --- | --- |
| **Number** | 12 | 52 |  |  |
| **Median age in years** | 35(32.75, 37.75) | 35.31±5.99 | 0.637 |  |
| **dRVVT ratio** | 1.85±0.81 | 1.03(0.95, 1.15) | **<0.0001** | 0.92-1.11 |
| **SCT ratio** | 2.29±1.09 | 1.11(0.98, 1.22) | **<0.0001** | 0.84-1.16 |
| **IgG/IgM aCL positivity** | 12 (100%) | 28 (53.85%) | **0.0029** | Negative (<20 UI) |
| **IgG/IgM aβ2GPI positivity** | 12 (100%) | 17 (32.69%) | **<0.0001** | Negative (<20 UI) |
| **History of thrombosis** | 3 (25%) | 12 (23.08%) | 0.8873 |  |
| **History of adverse pregnancy delivery** | 10 (83.33%) | 47 (90.38%) | 0.4805 |  |
| **PLT (*10^9/L)** | 163(131, 202) (N=11) | 215±64.95 (N=45) | **0.0449** | (125-350) *10^9/L |
| **C3 (g/L)** | 0.71±0.15 (N=11) | 0.87(0.79, 0.97) (N=42) | **0.0022** | (0.85-2) g/L |
| **C4 (g/L)** | 0.16±0.05 (N=11) | 0.23(0.19, 0.29) (N=43) | **0.0009** | (0.12-4) g/L |

**PAPS, primary APS.**
